# Synergistic Antimicrobial Effects of Silver/Transition-metal Combinatorial Treatments

**DOI:** 10.1038/s41598-017-01017-7

**Published:** 2017-04-18

**Authors:** Javier A. Garza-Cervantes, Arturo Chávez-Reyes, Elena C. Castillo, Gerardo García-Rivas, Oscar Antonio Ortega-Rivera, Eva Salinas, Margarita Ortiz-Martínez, Sara Leticia Gómez-Flores, Jorge A. Peña-Martínez, Alan Pepi-Molina, Mario T. Treviño-González, Xristo Zarate, María Elena Cantú-Cárdenas, Carlos Enrique Escarcega-Gonzalez, J. Rubén Morones-Ramírez

**Affiliations:** 1 Universidad Autónoma de Nuevo León, UANL, Facultad de Ciencias Químicas, Av. Universidad s/n, Cd, Universitaria, 66451 San Nicolás de los Garza, NL Mexico; 2 Centro de Investigacion en Biotecnologia y Nanotoxicologia, Facultad de Ciencias Quimicas, Universidad Autonoma de Nuevo Leon, Parque de Investigacion e Innovacion Tecnologica, Km. 10 autopista al Aeropuerto Internacional Mariano Escobedo, Apodaca, Nuevo Leon 66629 Mexico; 3 Centro de Investigación y de Estudios Avanzados del IPN, Unidad Monterrey, Parque PIIT, 66600 Apodaca, Nuevo León Mexico; 4 Cátedra de Cardiología y Medicina Vascular, Escuela de Medicina, Tecnologico de Monterrey, Monterrey, Nuevo León Mexico; 5 Centro de Investigación Biomédica, Hospital Zambrano-Hellion, Tecnologico de Monterrey, San Pedro Garza-García, Nuevo León Mexico; 6 Departamento de Microbiología, Centro de Ciencias Básicas, Universidad Autónoma de Aguascalientes, Av. Universidad 940, Colonia Ciudad Universitaria C.P., 20131 Aguascalientes, Aguascalientes Mexico; 7 Universidad Autónoma de Nuevo León, UANL, Facultad de Ciencias Biológicas, Av. Universidad s/n, Cd. Universitaria, 66451 San Nicolás de los Garza, NL Mexico; 8 Universidad Autónoma de Nuevo León, UANL, Facultad de Ingeniería Mecánica y Eléctrica, Av. Universidad s/n, Cd. Universitaria, 66451 San Nicolás de los Garza, NL Mexico

## Abstract

Due to the emergence of multi-drug resistant strains, development of novel antibiotics has become a critical issue. One promising approach is the use of transition metals, since they exhibit rapid and significant toxicity, at low concentrations, in prokaryotic cells. Nevertheless, one main drawback of transition metals is their toxicity in eukaryotic cells. Here, we show that the barriers to use them as therapeutic agents could be mitigated by combining them with silver. We demonstrate that synergism of combinatorial treatments (Silver/transition metals, including Zn, Co, Cd, Ni, and Cu) increases up to 8-fold their antimicrobial effect, when compared to their individual effects, against *E. coli* and *B. subtilis*. We find that most combinatorial treatments exhibit synergistic antimicrobial effects at low/non-toxic concentrations to human keratinocyte cells, blast and melanoma rat cell lines. Moreover, we show that silver/(Cu, Ni, and Zn) increase prokaryotic cell permeability at sub-inhibitory concentrations, demonstrating this to be a possible mechanism of the synergistic behavior. Together, these results suggest that these combinatorial treatments will play an important role in the future development of antimicrobial agents and treatments against infections. In specific, the cytotoxicity experiments show that the combinations have great potential in the treatment of topical infections.

## Introduction

In the past three decades, global usage of antibiotics has become disproportionate, and, in the majority of instances, unsuitable^1^. This has led to the emergence of multi-drug resistant strains. Highly resistant Gram-negative bacteria, such as *P. aeruginosa*, *Acinetobacter*, *Klebsiella* and *Escherichia* species, have become worldwide relevant pathogens^2^ and are part of the ESKAPE list which includes the most current deadly pathogens^3^. In addition, some Gram-positive bacteria, such as methicillin-resistant *Staphylococcus aureus* ^1, 4, 5^, and vancomycin-resistant *Streptococcus pneumonia* ^4^, are pathogens also very difficult to treat. The spreading of antibiotic-resistant strains embodies an issue in which international health institutions spend millions of dollars annually^5^. As a result, increasingly significant attention has been paid to the development of novel antibiotics; particularly metallo-pharmaceuticals, which are compounds that involve metals due to their therapeutic action, and currently belong to a class of promising antimicrobial compounds aimed to overcome resistant strains^6–13^.

Transition metal species, and remarkably silver compounds, lie among the most studied metallo-pharmaceuticals^14, 15^. Silver compounds have been used as antimicrobial agents since ancient times. It has been reported that Greek and Roman civilizations used silver as a disinfectant for water and food, as well as a wound-healing enhancer. Moreover, silver species had been widely applied in traditional Chinese medicine^6–10^, and lately, as disinfectants for water, fruits, and vegetables in several Latin American countries^16^. Hereafter, during the last decade, several health supplies, such as textile materials^17, 18^, implants, catheters, and gauze pads^7, 8^, have made extensive use of silver species and engineered delivery systems involving silver derivatives as antimicrobial agents^6, 7, 10, 16^. Recently, these antimicrobial developments have been focused to combat multi-drug resistant bacteria^19^. Nonetheless, silver is not the only transition metal widely used as an antimicrobial agent; zinc^20, 21^ and copper^22^ species are known to also exhibit antimicrobial effects. More interestingly, these metals are also considered as essential metals since they have been found to play key roles in many cellular processes^23^. However, even essential metals, when present in relatively high concentrations, become harmful^24–27^.

In bacteria, metal ions can inhibit growth completely at mM nominal concentrations^28^. In different *E. coli* strains this effect has been observed at >1 mM of total Co, and for sulfur-oxidizing bacteria isolated from concrete, complete growth inhibition can be observed at 5 mM Ni^29^. In addition, ROS stress responses have been observed at lower concentrations when using CuO nanoparticles with an *E. coli* biosensor at 0.1 mM Cu^30^. However, it is a well-documented fact that one of the main limitations in using transition metals as therapeutic agents is their toxicity to eukaryotic cells. The cytotoxicity of transition metals has been reported in the literature for human gingival fibroblast (HGF)^31^, TC_50_s of 78.7, 344, 705.8 and 872.9 µM, for Cd, Cu, Co, Ni respectively, and for HeLa cells^32^ the TC_50_ of Zn has been reported to be 600 µM.

To overcome the cytotoxicity drawbacks of using transition metals as antimicrobial agents, it has been suggested that the overall minimum inhibitory concentration can be lowered by the formulation of proper combinations of metals, resulting in effective therapies under the toxicity threshold^10, 33^. In this work, we hypothesized that this approach was suitable for silver/transition metal combinations, and used *Escherichia coli* and *Bacillus subtilis* as the Gram-negative and Gram-positive models, respectively, to test the synergistic effect of these combinatorial treatments. Our results support the effectiveness of this approach. When combinatorial treatments, composed of transition metals (zinc, copper, nickel, or cadmium) and silver, are tested against *E. coli* and *B. subtilis*, the combinations exhibit up to an 8-fold enhancement in their antimicrobial properties, compared to the individual treatments. We analyzed the combined antimicrobial effects and found them to be synergistic against *E. coli* and *B. subtilis*. Furthermore, we show that the minimum inhibitory concentrations of the transition metals are lowered significantly. We assessed the cytotoxicity of the treatments and found low cytotoxic effects for most combinatorial silver-transition metal treatments in three different cell lines (blasts and melanoma rat cell lines and a human keratinocyte cell line). The latter as a representation of the major cell line type found in the epidermis, which is reported to be a promising screening tool for predicting irritant and toxic skin effects^34^.

## Materials and Methods

### Minimum Inhibitory Concentration

Minimum Inhibitory Concentrations (MICs) were assessed in 96-well plates (Costar, Corning) based on a modified methodology reported by Andrews^35^ and NCCLS^36^. Stocks of transition metal salts, NiSO_4_·6H_2_O, CoCl_2_·6H_2_O, 3CdSO_4_·8H_2_O, CuSO_4_·5H_2_O, ZnSO_4_·7H_2_O, were prepared at final concentration of 100 mM using distilled water as the vehicle. A stock solution of 5 mM AgNO_3_ was also prepared under similar conditions. To perform the MIC tests, we added in the top well the necessary volume from our stocks to reach a 16 mM concentration of the transition metal within a final volume of 200 µL. Next, serial dilutions were performed by taking 100 µL from every next well into 100 µL of culture media and discarding the last 100 µL from the last dilution. This way, the tested concentrations ranged from 8 to 0.0156 mM for the transition metals after the bacteria inoculum is added. A similar procedure was performed with the silver stock, where the top well was prepared to achieve a final concentration of 3840 µM, after addition of the inoculum. The nominal concentrations of Ag ranged from 1920 to 3.75 µM.

To inoculate each test well in the MIC assay, an overnight culture (20 hours culture incubated at 37 °C–150 rpm) for each strain (*Escherichia coli*, ATCC-11229, or *Bacillus subtilis* ATCC 23857) was diluted 1:250 in fresh medium and incubated until a critical optical density (OD_600_ of 0.2 ± 0.02) was reached; adjusting with fresh medium if necessary in order to reach a cell concentration range between 10^7^–10^8^ cells/mL. Cell number was monitored by the plate-counting method measured by serial dilutions. From this, a 1:100 dilution was made with fresh medium in a 1.5 mL tube, then 100 µL of this dilution was added to each test well in order to achieve a final concentration of 10^5^ cells/mL, following incubation at 37 °C–150 rpm.

After 20 h of incubation at 37 °C, the ODs of the control and the treated inoculum were measured. We next determined that the MIC for each of the transition metals corresponded to the concentration at which no significant growth was observed (OD_600_ < 0.05). All tests, and their respective control samples, were performed in replicates of 3.

### MIC determination through Checkerboard assays for the silver/transition metal combinations (STMCs)

Checkerboard assays^37, 38^ were performed in 96-well polystyrene plates, in order to unveil the synergistic effects of silver and transition metals. The assays tested the MIC of each transition metal and the STMCs corresponding to 0, 0.5, 0.25, and 0.125 of their respective individual MIC (Supplementary Figure S1). The MIC fractions of silver were prepared along the abscissa axis of the plate and the transition metal MIC fractions were placed along the ordinate axis of the plate. Concentrated metal solutions were prepared in culture media, so that when added to the culture the needed volume of metal and inoculum MICs fractions was reached.

Bacterial cultures were grown for 16 h at 37 °C and 150 rpm, from which a dilution (1:250) was prepared in fresh Luria Bertani broth (LB) medium, and incubated until it reached a critical optical density (OD_600_ = 0.20 ± 0.02), adjusting with fresh medium if necessary. This allowed us to obtain a concentration range between 10^7^–10^8^ cells/mL. A 1:20 dilution of this culture was adjusted in LB medium, followed by the addition of 20 µL (1:10) of the inoculum, this resulted in an estimated concentration of ~10^5^ cells/mL within a final volume of 200 µL. The 96-well plates were incubated at 37 °C and 150 rpm for 20 h. After incubation, the ODs of the control and the treated inoculums were measured, and the respective values were recorded. Each STMC, and their corresponding control samples, were tested in triplicates per plate.

STMCs at lower concentrations were made to emulate an isobologram analysis^39, 40^ in 96-well plates. MIC fractions of 0.25, 0.125 and 0.062 of silver and each transition metal (M) nominal concentrations were combined to achieve final ratios of: 0.062:0, 0.062:0.25, 0.062:0.125, 0.062:0.062, 0.125:0.062, 0.25:0.062, 0:0.062 of Ag:M in a final volume of 200 µL, including the bacteria inoculum. These combinations were diluted 2-fold and 100 µL were transferred each time after a thorough mixing and discarding the last 100 µL from the 5^th^ dilution.

The inoculation of these wells was performed using the same procedure described in the MIC section. The 96-well plates were incubated at 37 °C and 150 rpm for 20 h. After incubation, the ODs of the control and the treated inoculums were measured, and the respective values were recorded. Each STMC additional dilutions, and their corresponding control samples, were tested in triplicates.

### Antibacterial effect of STMCs

One STMC per transition metal used was selected to assess its bactericidal activity at 1 h. The antimicrobial effect was tested in an overnight culture of *E. coli* ATCC 11229 that was diluted (1:250) in 250 mL of LB medium, followed by incubation at 37 °C-150 rpm until it reached exponential phase at a critical optical density (OD_600_ = 0.10–0.20). From this medium, 25 mL were transferred to 250 mL flasks, with the addition of their corresponding amount of STMC solution, and incubated at 37 °C and 150 rpm for 1 h. This time point was chosen in order to observe if there was a short-time exposure bactericidal effect. After incubation CFU/mL were determined through the drop-count method. 10-fold diluted aliquots were obtained and, three 10 µL drops of each of the diluted samples were placed on LB agar plates. The plates were incubated at 37 °C during 24 h and at that time the viable bacterial colonies were counted. All tests, and their respective control samples, were performed in replicates of three.

### Flow cytometry analysis

STMCs were prepared as described in the checkerboard methodology. The bacteria inoculum was incubated at 37 °C and 150 rpm for 20 h. After the incubation time, the bacteria were chilled on ice until they were analyzed. Flow cytometry with a propidium iodide dye was used to detect differences in membrane permeability^10^. A double-stain procedure with two fluorescent nucleic acid stains was followed as previously described^41^. Briefly, 10^6^ bacteria were directly labeled and incubated (15 min, dark, room temperature) with propidium iodide 4 μg (PI, Sigma Aldrich, St. Louis, MO., USA) and SyBR-G 1X (Life Technologies, Carlsbad, CA., USA) in 250 μL PBS. For calibration purposes a sample of each bacteria was heated for 10 min at 96 °C as a positive control. Analysis was performed in a BD FACS Canto II cytometer (BD, San Jose, CA., USA) configured with channel PerCP (670 LP nm band-pass filter) and FITC (530/30 nm band-pass filter) for PI and SyBR-G fluorescence acquisition, respectively. A total of 10,000 events per sample were collected at low flow rate. Signals were collected in a logarithmic scale in FSC and SSC and bacteria were double discriminated on green (live) and red (dead-permeable) fluorescence; double negative signals (debris) were also evaluated. Data were analyzed with the FlowJo software v.8.8.6 (Tree Star, Inc., Ashland, OR., USA) and the experiment was independently replicated three times.

### Cytotoxicity assay in mammalian cells

HaCaT cells, a spontaneously transformed human keratinocyte cell line, (kindly provided by Dr. Pablo Ortiz, CIBO, Mexico) were cultured in DMEM medium supplemented (sDMEM) with 1 g/L glucose, 4 mM L-alanyl-glutamine, 10% heat-inactivated fetal bovine serum (FBS; Gibco, NY, US), 50 IU/penicillin and 50 μg/ml streptomycin (Sigma, Israel) at 37 °C and 5% CO_2_. To measure mitochondrial activity as a viability marker, the MTT assay was performed in triplicates, as described previously^42^ with little modifications. To expose the cultures to the individual metals and the STMC treatments, 96-well microplates were seeded with 4 × 10^4^ cells in 100 μl of sDMEM and left to attach overnight. Next, the old medium was replaced with 200 μl of sDMEM previously formulated with the different concentrations of transition metals. 10% DMSO was used as a positive control and medium alone as the negative control. After a 24 h exposure, the medium was discarded and 100 μl of fresh MTT solution (0.5 mg/ml of MTT in DMEM medium without FBS) was added to the cells and they were incubated for 4 h at 37 °C and 5% CO_2_. The MTT solution was substituted with 100 μl of Isopropanol-0.04 N HCl, in order to dissolve the formazan crystals and the optical density (OD_595nm_) (655 nm in the reference) was measured in an iMark microplate reader (Bio-Rad, Tokyo, Japan). Viability was calculated as the ratio of the mean between the OD_595nm_ of the treated groups and the OD_595nm_ of the negative control.

H9c2 cells (Rat cardiomyoblast, CRL-1446, ATCC, Manassas, VA, USA) were maintained at 37 °C in a 5% CO_2_ humid atmosphere in 25 cm^2^ culture flasks in DMEM media supplemented with 10% fetal bovine serum (FBS), penicillin (100 U/mL) and streptomycin (100 µg/mL). Cytotoxicity of transition metals was analyzed on H9c2 cells. Briefly, once H9c2 cells reached 70–80% confluence in the 25 cm^2^ flasks, cells were plated into 96 wells trays at a density of 2 × 10^3^ cells per well in 100 µL medium. Mouse skin melanocytes (B16F10 cells, CRL-6475, ATCC, Manassas, VA) were maintained at the same culture conditions described above, except that they were in a DMEM/F12 culture medium supplemented with 10% FBS and 1X antibiotic-antimitotic (all from ThermoFisher Scientific, Waltham, MA) and plated into 96 wells plates at a cell density of 5 × 10^3^ cells per well in 100 µL of medium. For these cell lines, twenty-four hours after they were plated, the different treatments were added and then the treated cells were incubated for 48 h. Cytotoxicity was assessed, for both cell lines, by the AlamarBlue® assay (Invitrogen, Eugene, OR) according to the manufacturer instructions. The half maximal inhibitory concentration (IC_50_) was determined using the software Origin (Northampton, MA), as previously reported^43^. All experiments, and their respective controls, were performed in triplicates.

### Data analysis and Calculations of Free Ions in Solution

To estimate the significance of the observed differences between the treatments employed, all collected data was subjected to an analysis of variance (ANOVA) and Fisher’s least significant difference (LSD) tests, using Microsoft Excel 2013.

The interaction of silver with transition metals was analyzed using the Bliss independence model described by Hegreness *et al*.^4^, which states that the interaction can be considered as synergistic when the combined effect of the antimicrobial agents is greater than the predicted effect of its individual components. Therefore, the *S* value; the difference between the predicted value of individual components *x* and *y* (*f*_*x0*_ and *f*_*0y*_, respectively) and the combined treatment *xy* (*f*_*xy*_) is denoted in the form:

1 $$S=(\frac{{f}_{x0}}{{f}_{00}})(\frac{{f}_{0y}}{{f}_{00}})-\,\frac{{f}_{xy}}{{f}_{00}}\quad \quad {\rm{Bliss}}\,{\rm{Independence}}\,{\rm{Model}}$$

where: *f*_*x*0_ = treatment with Ag, *f*_0*y*_ = treatment with the transition metal, *f*_*xy*_ = treatment Ag + metal, *f*_00_ = growth control of a culture that has not been treated with the transition metals. The value of S describes the interaction between combinatorial treatments as follows: S > 0 Synergistic; S = 0 Additive; S < 0 Antagonistic.

Speciation for metals and STMCs was performed using the Visual MINTEQ 3.1 program designed and developed by Jon Petter Gustafsson at KTH in Sweden^44^. Metal species were calculated using medium formulations as reported by manufacturers (BD, ThermoFisher Scientific, Sigma-Aldrich) at 37 °C, and pH of 6.8 and 7.0 for LB and DMEM mediums, respectively.

## Results and Discussion

### Microbial Toxicity of Transition Metals in Prokaryotic Cells

Since the times of the first human civilizations, society has taken advantage of the antimicrobial properties exhibited by different metals^22, 45, 46^. Nowadays, there is a wide variety of compounds and materials that exhibit antimicrobial activity due to their transition metal content, such as Ag, Zn and Cu^21^. We here hypothesize that treatments involving silver salts and transition metal combinations (STMCs) will exhibit synergistic antimicrobial effects, when compared to the effect of the individual compounds. To evaluate the properties of the different STMCs, we analyzed the synergistic effects of antimicrobial activity in *E. coli* (Gram-negative microorganism) and *B. subtilis* (Gram-positive microorganism).

We first tested Zn, Cd, Ni, Co, Cu and Ag at different concentrations to determine their individual MIC values. The summary of the observed MIC values is displayed in Table 1. Most of the metals tested in *E. coli*, Zn, Cd, Ni, Co, and Cu, Ni, Co and those in the assays for *B. subtilis* have MICs between 1 and 2 mM. Only Cu, with an MIC of 4 mM in *E. coli* assays, and Ag with an MIC of 60 µM for both microorganisms, were found apart from the range previously mentioned. All of these MICs correlate with those found in the literature^10, 47, 48^. Moreover, the available free metal ions in the medium may vary due to its composition; therefore, total free ions were calculated and are reported in parenthesis in Table 1 and also in Supplementary Tables S1 and S2. As can be observed in Tables 1, S1 and S2, the percentage of the total metal available as free ions varies depending on the metal and its concentration.

**Table 1:** Nominal (Free Ion) Minimum Inhibitory Concentrations of Individual Transition Metals.

Transition Metal	*E. coli* Nominal (Free ion)	*B. subtilis* Nominal (Free ion)
Ag	60 µM (22.8 nm)	60 µM (22.8 nm)
Zn	2 mM (980.1 µM)	0.25 mM (118.7 µM)
Cd	2 mM (229.3 µM)	0.015 mM (0.16 µM)
Ni	2 mM (531.6 µM)	2 mM (531.6 µM)
Cu	4 mM (163.9 µM)	2 mM (2.4 µM)
Co	1 mM (656.4 µM)	1 mM (656.4 µM)

MICs for each of the individual transition metals and Ag were determined under identical culture conditions.

### Synergistic effect of silver/transition metal STMCs

**Figure 1:**
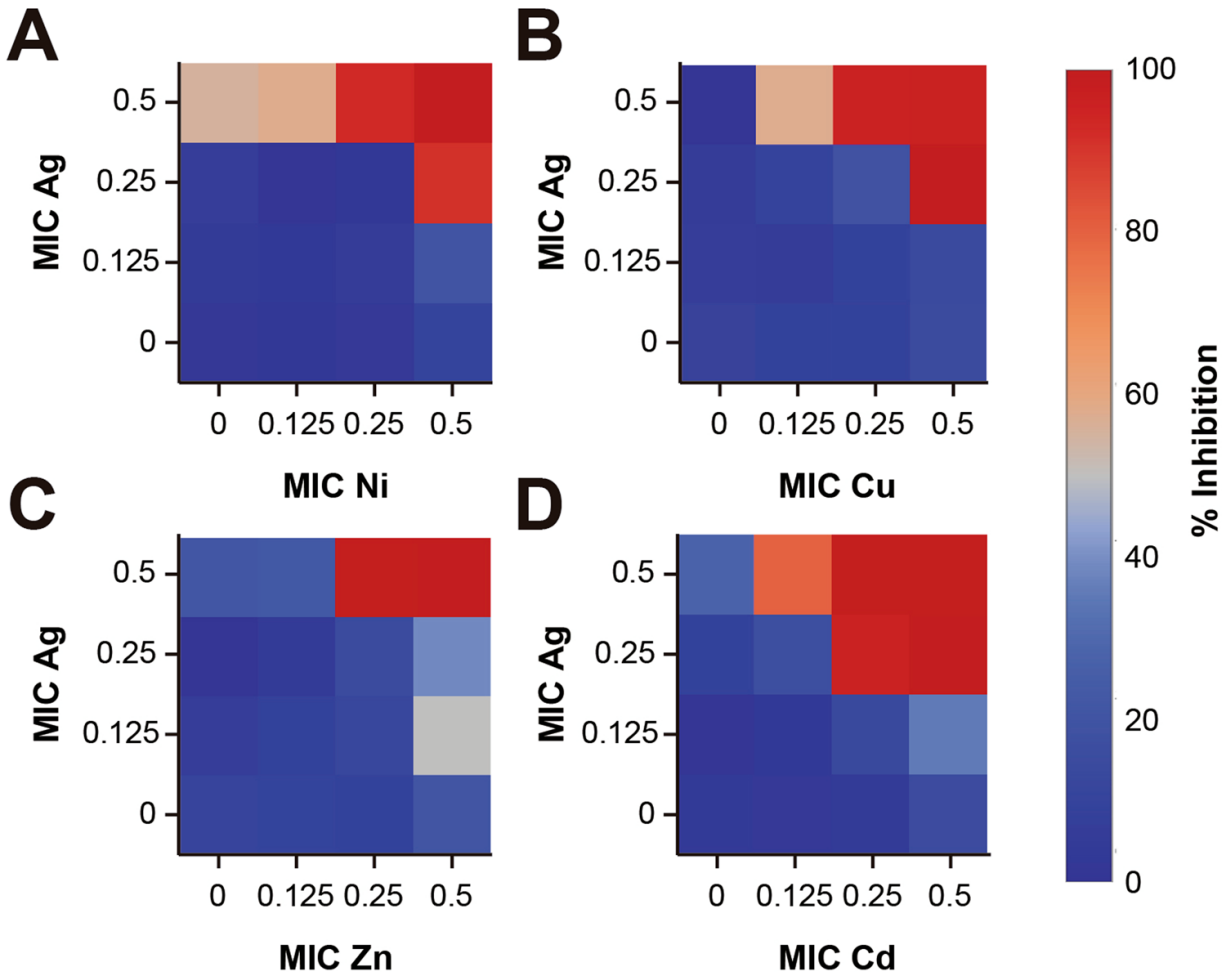
Inhibitory Effect of the MIC dilutions of Ag potentiated by transition metals against *E. coli*. Inhibitory percentage by sub-inhibitory combinations of Ag-transition metals in *Escherichia coli* ATCC 11229. Experiments with combinatorial treatments of Ag with (**A**) Ni, (**B**) Cu, (**C**) Zn and (**D**) Cd. Each combinatorial treatment with inhibition >80% is significantly different to the effects of the individual treatments. Each checkerboard treatment was performed in triplicates.

We tested combinations of Ag with different transition metals (Cu, Co, Zn, Ni and Cd) via a checkerboard assay, a widely used methodology to test synergistic effects between antimicrobial agents^38, 49–51^. The inhibitory effect of the different transition metal combinations with silver against *E. coli* is shown in Fig. 1. It can be observed that a reduction to 0.25 of the MIC of silver is achieved when combined with Zn, Cd, Ni, and Cu. Moreover, a reduction to 0.5 of the MIC of each of the transition metals is shown, respective to the values when tested alone (Fig. 1A–D). Cd, Ni, and Cu were able to enhance the antimicrobial activity of silver at both: 0.5 and 0.25 of their individual MICs (Fig. 1A,B,D), whereas similar behavior was observed in the case of Zn, when present at 0.5 of its MIC, albeit in a lower extent (Fig. 1C). All the STMCs, with inhibitory activities above 80%, exhibited statistically significant differences when compared to the treatments with individual transition-metal salts (p < 0.05). Only the STMCs made with Co were not able to increase the inhibitory effect of the silver MIC fractions.

**Figure 2:**
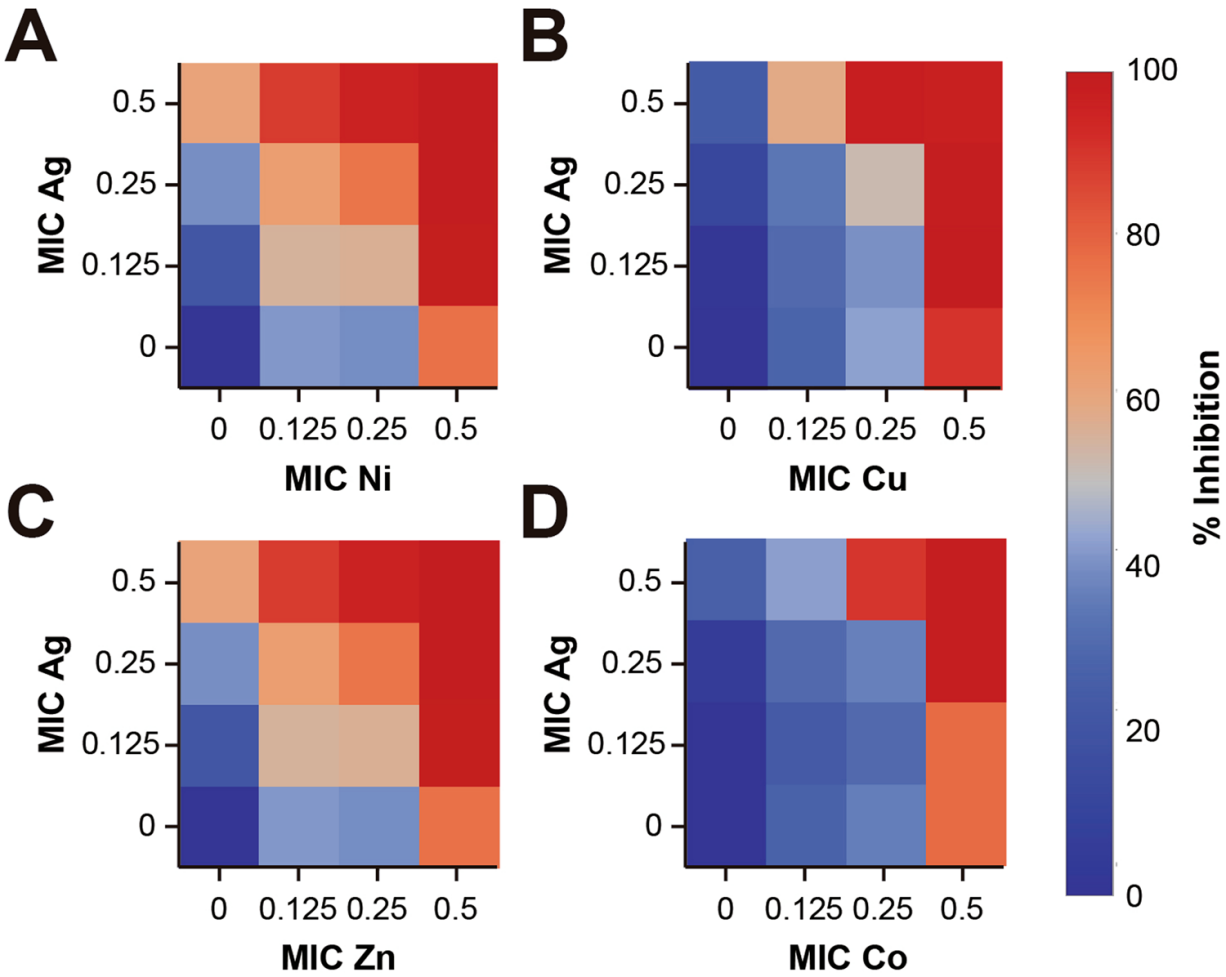
Inhibitory Effect of the MIC dilutions of Ag potentiated by transition metals against *Bacillus subtilis*. Inhibitory percentage by sub-inhibitory combinations of Ag-transition metals in *Bacillus subtilis* ATCC 23857. Experiments with combinatorial treatments of Ag with (**A**) Ni, (**B**) Cu, (**C**) Zn and (**D**) Co. Each combinatorial treatment with inhibition >80% is significantly different to the effects of the individual treatments. Each checkerboard treatment was done in triplicates.

Figure 2 shows that the transition metal that best enhanced the inhibitory activity of Ag against *Bacillus subtilis* was Zn, since it decreased its MIC to 0.125 and 0.25 when combined with 0.5 of the MIC of Ag. For the rest of the metals, when combining 0.5 MIC of each individual transition metal, the experiments show that >80% inhibition can be achieved with each of the fractional Ag MICs tested. For the case of Cu the data shows that at 0.5 and 0.25 of the Cu MIC, the MICs of Ag can be decreased four and two-fold, respectively, to achieve >80% inhibition in *B. subtilis*. Ni and Co at 0.5 MIC can enhance by two-fold the inhibitory effect of Ag. The calculated free ions of the STMCs tested in both bacteria are reported in Supplementary Tables S3 (*E. coli*) and S4 (*B. subtilis*).

The results described here have also been observed with different transition metals in the form of complexes against Gram-positive bacteria^52^ and fungi^53, 54^. In this work, we observed that four of the five metals used, when combined with silver, were able to increase the inhibitory effect of silver MIC fractions in *E. coli*. Only Co did not show this effect significantly. Similarly, in the assays made with *B. subtilis*, four of the five metals were able to increase the effects of Ag. Only for the case of Cd we did not observe the phenotype. Previously, different reports have used transition metals (primarily in nanoparticle or polymeric systems) in combination with silver to take advantage of its inhibitory effects; such is the case of Cu^55^, Zn^56^ and Co^57^.

**Figure 3:**
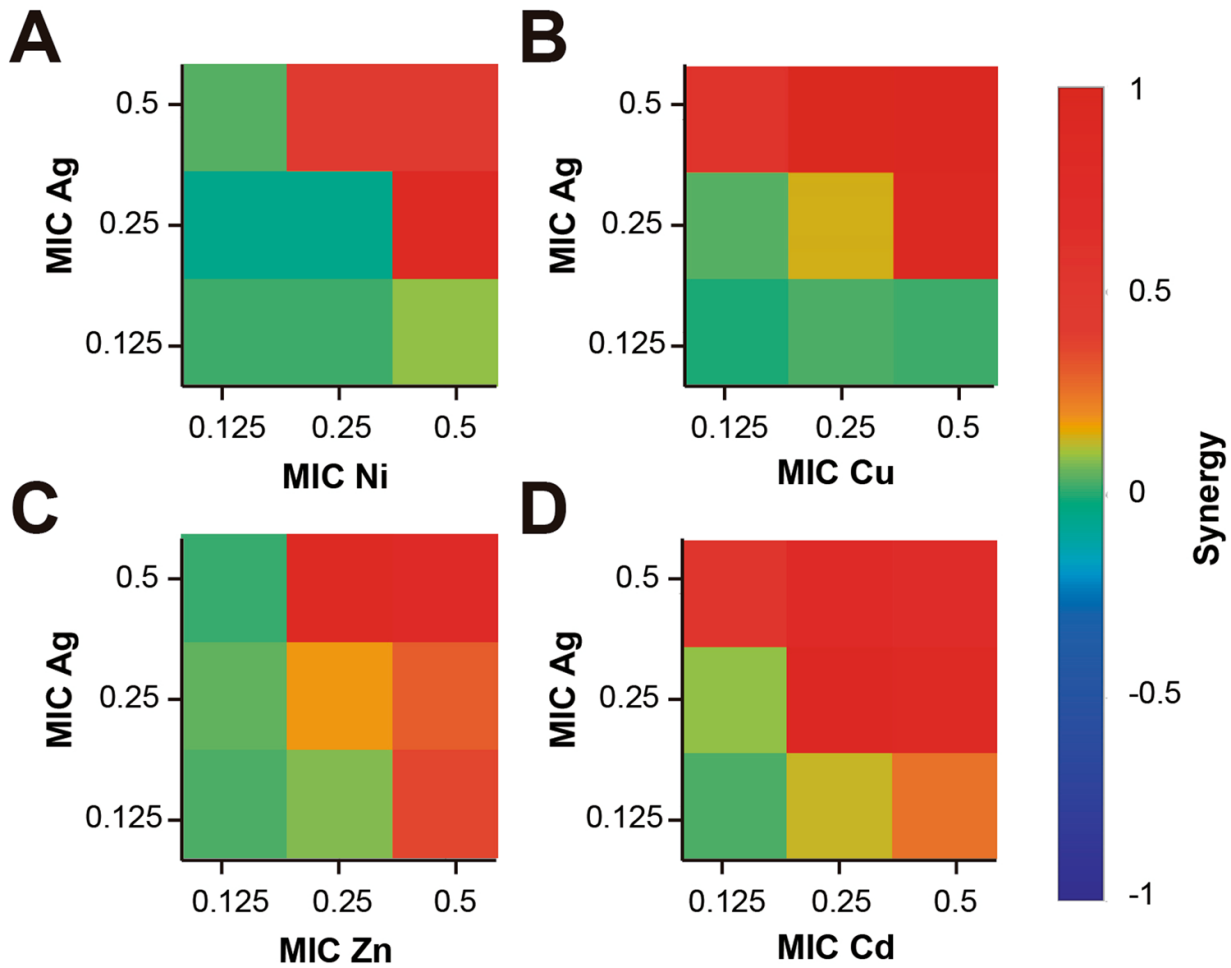
Analysis of Ag-Transition Metal Interactions. Classification of the different interactions between Ag-transition metal in *Escherichia coli* ATCC 11229. The interactions of Ag with (**A**) Ni, (**B**) Cu, (**C**) Zn and (**D**) Cd, are classified as synergistic, additive or antagonistic, value >0, =0 and <0, respectively. Each checkerboard treatment was done in triplicates.

The nature of the interactions between the components of the STMCs, in virtue of their antimicrobial effect against *E. coli*, were assessed as a function of their individual and combined activities using the Bliss Mathematical Model represented in Equation 1. These results are displayed in Fig. 3 and they show that the overall response appears to be synergistic. Even in the samples with inhibitory activities below 80%, the effect was slightly higher than the individual counterparts (Fig. 3A–D). In the case of Ni and Cu, an antagonistic interaction with Ag can be observed (Fig. 3A,B), however, this behavior is not present when one of the concentrations of the components increases, particularly in Ni, which exhibits synergistic effects when the concentration of Ag is found at 0.5 of its MIC (Fig. 3A).

**Figure 4:**
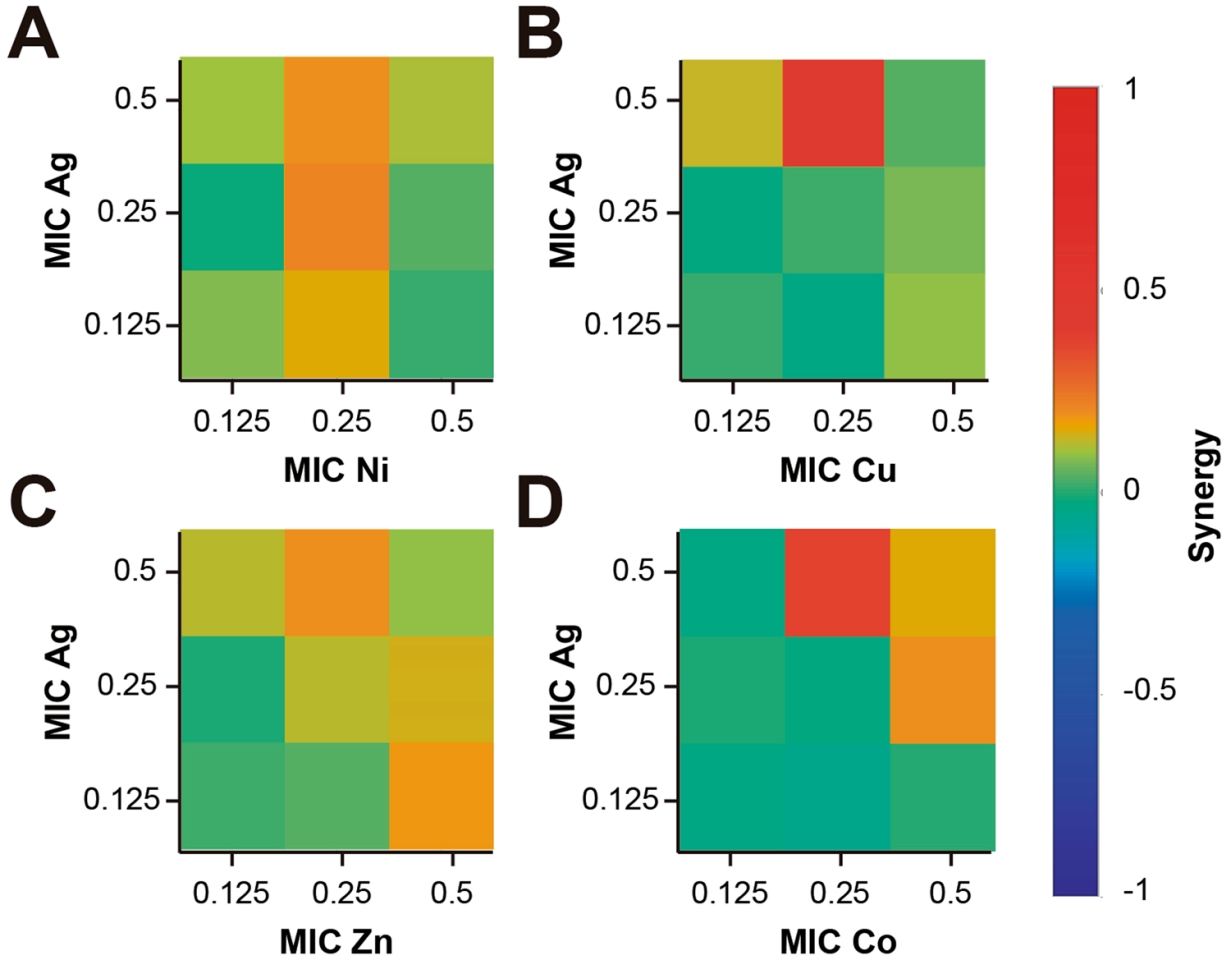
Analysis of Ag-Transition Metal Interactions. Classification of the different interaction between Ag-transition metal in *Bacillus subtilis* ATCC 23857. The interactions of Ag with (**A**) Ni, (**B**) Cu, (**C**) Zn and (**D**) Co, are classified as synergistic, additive or antagonistic, value >0, =0 and <0, respectively. Each checkerboard treatment was done in triplicates.

Figure 4 shows the interactions between components in the *B. subtilis* assays. The interaction between Ag-Ni can be classified as synergistic, especially in the combinations of 0.125, 0.25 and 0.5 MIC of Ni with a 0.25 MIC of Ag. However, we can see that in combinations with high concentrations of Ag the interaction is antagonistic, changing with the increase of Ni. In the same manner, at low concentrations of Ag and Cu the interactions are also antagonistic, but they switch to synergistic with combinations of 0.25 and 0.5 MIC of Ag with 0.5, 0.25 and 0.125 MIC of Cu. The same synergistic effect can be observed with Ag-Zn at 0.25 and 0.5 MIC of Ag with 0.5, 0.25 and 0.125 MIC of Zn. With Co the only combinations that can be classified as synergistic are 0.5 MIC of Ag with 0.5 MIC of Co and 0.5 MIC of Ag with 0.25 and 0.5 of Co.

To explore a possible hormesis effect, exhibited in some biological systems where effects (such as growth) are accentuated in the presence of low concentrations of a compound^58^, we performed treatments at lower concentrations of the STMCs. The results demonstrated that no significant inhibitory or enhanced growth effects where shown for most of the STMCs. Only for the case of Ag-Ni and Ag-Co a slight hormesis effect was observed in *B. subtilis*. (Supplementary Tables S5 and S6).

Even though there is a wide array of literature focused on combination of antimicrobial agents and their enhanced effects as a combined treatment, the nature of the interaction of the combined components has not been widely explored. Different works have reported conditions where both synergistic and antagonist effects have been observed between Ag-Cu, the interaction depends on the bacterial species treated^59^. In this work, it can be observed that for the case of Gram-negative *E. coli*, most of the STMCs showed strong synergistic effects. In Gram-positive *B. subtilis*, the same combinations, although exhibited synergistic behavior, most of them are closer to an additive behavior. As can be seen in Figs 3 and 4, most of the synergistic interactions are found at combinations between the higher MIC fractions, and as the MICs fractions decrease, the interactions shift to an antagonist nature. This effect, although stronger for our experiments with Gram-positive bacteria, was also observed in our assays with Gram-negative bacteria. A very similar behavior has been previously reported for combinations with Ag-Cu tested in *L. pneumophila* ^60^. In other cases, as shown in Fig. 4, an STMC can exhibit synergistic behavior, but when the concentration of one of the components decreases the synergistic effects increases. A reason for this could be that, since some of the STMCs have similar cellular targets, when high concentrations are present one of the components could be acting in an overlapping target, but when lowering the concentration, both can exert its antimicrobial activity separately^27^.

### Enhanced bactericidal activity of Ag in silver/transition metal STMCs

**Figure 5:**
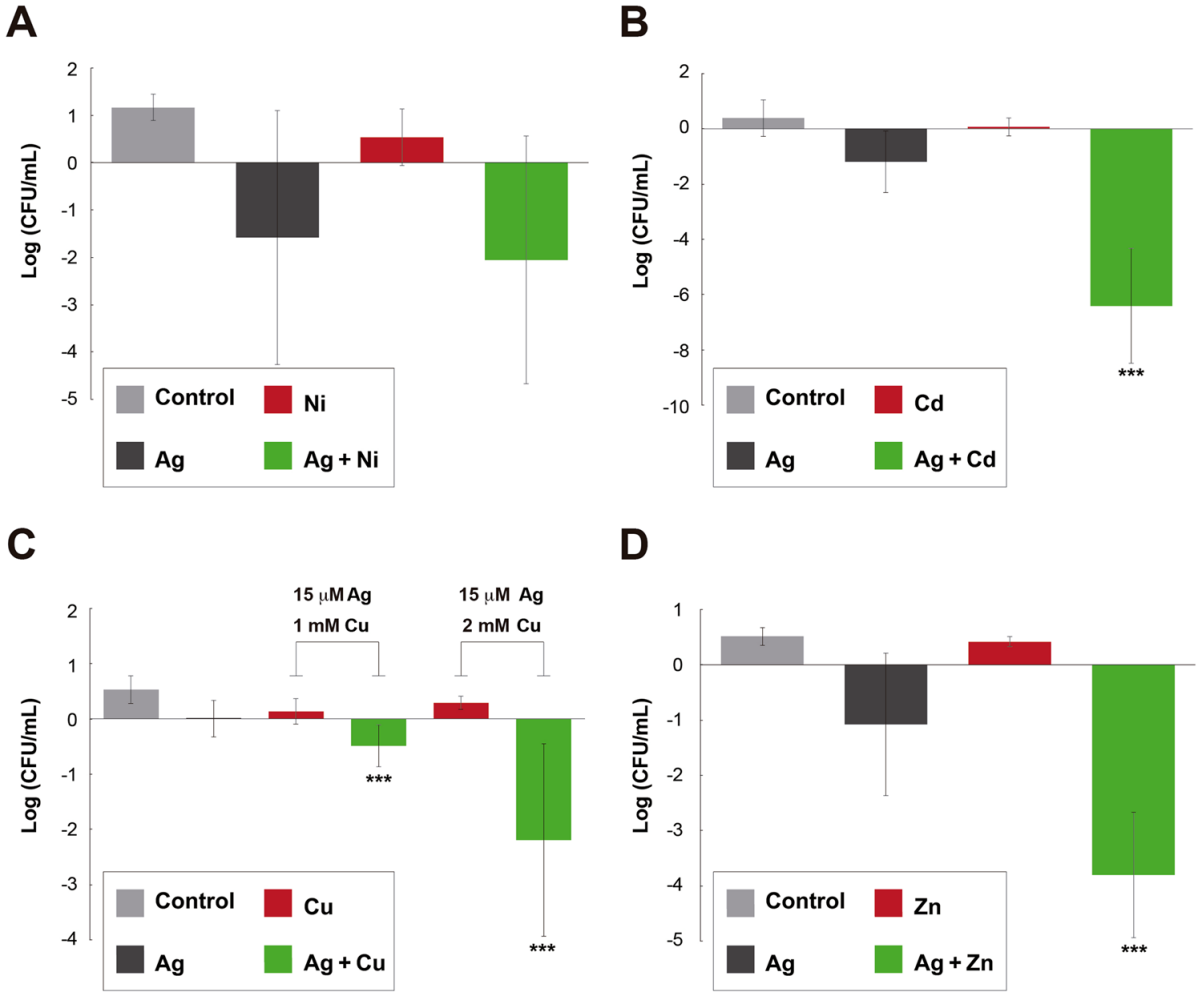
Bactericidal Effect of Ag Potentiated by Transition Metals. Log change in CFUs/mL with respect to time zero, in *Escherichia coli* ATCC 11229 after 1 hour treatment with: LB (control), Ag, specific transition metal and their combination. As nominal concentrations: (**A**) Ag 30 µM, Ni 0.5 mM and the combination; (**B**) Ag 30 µM, Cd 1 mM and the combination; (**C**) Ag 15 µM, Cu 1 mM and 2 mM, and the respective combinations; (**D**) Ag 30 µM, Zn 0.5 mM and the combination. ***Corresponds to a p < 0.05, tested with an ANOVA, that there is a difference with respect to the control and each of the individual treatments. Error bars correspond to the standard deviation from experiments performed in triplicates.

To determine the ability of each transition metal and the STMCs to kill both Gram-negative and Gram-positive bacteria, we studied their bactericidal effects by exposing exponentially growing bacteria to different treatments. Figure 5 provides a representation of the bactericidal activity found for each STMC in *E. coli*. Evidently, Cd, Cu and Zn (Fig. 5B–D) were found to significantly enhance the bactericidal activity of Ag against viable *E. coli* in exponential phase (p < 0.5). In addition, the measured data demonstrates that the enhanced activity of silver reduces cell concentration (CFU/mL) between 2 and 4-log, compared to the decrease associated with the individual Ag treatment. However, Ag-Ni combinatorial treatments exhibited activities resembling that of the individual Ag antimicrobial effect (Fig. 5A), even though Ni was found to enhance the inhibitory activity of Ag (Fig. 2A). The same assays were performed with *B. subtilis* and the inhibitory STMCs; however, the results show no significant enhanced bactericidal effect (Supplementary Figure S2A–D).

### Flow cytometry analysis

**Figure 6:**
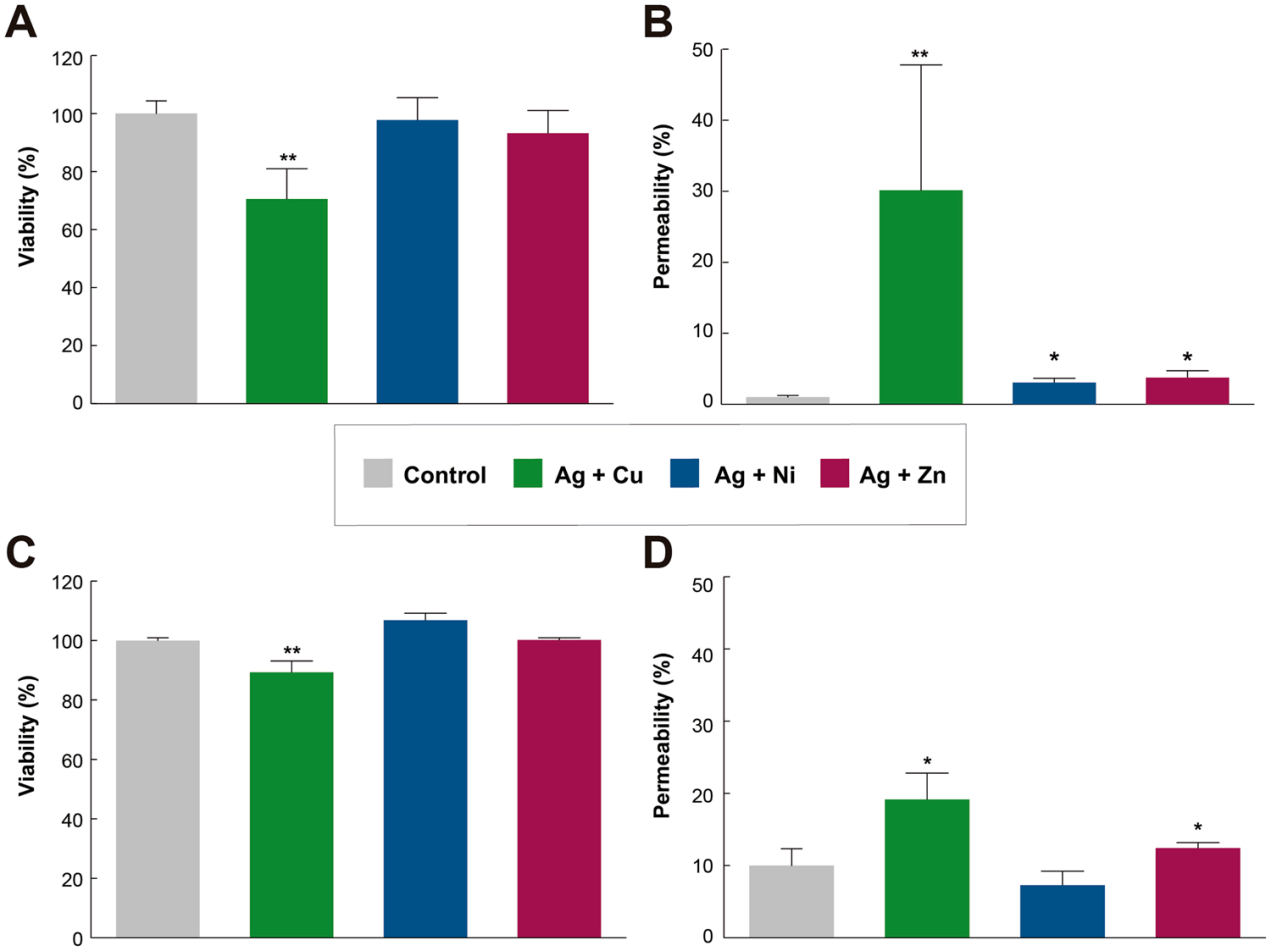
Viability and cell permeability assay of *B. subtilis* and *E. coli* treated with STMCs. Nominal concentrations of Ag/Cu Ag/Ni and Ag/Zn (15 μM/0.5 mM) by 24 h (37 °C). (**A**) SyBR-G *B. subtilis* positive cells represented as a measure of % of viability; whereas (**B**) *B. subtilis* PI positive cells are represented as a measure of % of permeability. (**C**) SyBR-G *E. coli* positive cells represented as a measure of % of viability; whereas (**D**) *E. coli* PI positive cells are represented as a measure of % of permeability Values represent mean ± SD (n = 3 experiments for each treatment). P values *P < 0.05 vs. Control, **P < 0.01 vs. Control.

The mechanism of antimicrobial synergy between silver and transition metals was explored by running cytometry assays using propidium iodide to determine changes in permeability. The Ag-Cu, Ag-Ni and Ag-Zn STMCs were selected since they showed inhibitory effects in both, Gram-positive and Gram-negative bacteria. At high Ag concentrations (30 μM) with the different transition metals at 1 mM (Cu, Ni, or Zn), cell death (Supplementary Fig. S3A,D) and lysis was clearly observed for both microorganisms. These STMCs also showed a high percentage of debris (Supplementary Fig. S3C,F) and in addition, an increased permeability was noticed (Supplementary Fig. S3B,E). Lower doses at half the lethal dose concentrations (Ag 15 μM) in combination with 0.5 mM (Cu, Ni, or Zn) show that across the range of concentrations, none or very slight cell death is observed for both *E. coli* (Fig. 6A) and *B. subtilis* (Fig. 6C). However, the permeability of the cells increases significantly between 20 to 50% for both microorganisms (Fig. 6B,D). Since bacterial membranes contain polymers with highly electronegative chemical groups, these can serve as sites for metal cation adsorption. Because of their ability to coordinate metals, it has been postulated that the membrane is the site at which some metals exert bactericidal toxicity. For Ag treatments, there are reports that show that Gram-negative and Gram-positive bacteria exhibit morphological changes, like the formation of a big cap between the cytoplasm membrane and the cell wall, causing serious damage on the latter^61^, as well as induction of OH^·^ production^10^. Additional works have also shown that Zn, as ZnO nanoparticles, can cause disruption of membrane morphology and induce oxidative stress^62^ in both Gram positive and Gram negative bacteria, similar to the effects shown with Ag treatments. On the other hand, Ni, Cu and Cd have been reported to cause disruption of biological processes within the cell, causing DNA alterations^63^, and increased lipid peroxidation^22, 64, 65^. Therefore, our results show that one possible mechanism of synergy between the STMCs, in both *B. subtilis* and *E. coli*, could be an initial increase in permeability and damage of the cell wall followed by disruption of different internal cellular processes.

### Toxicity Data for the Different Transition Metals

A data analysis was performed to compare the total MICs and the effective bactericidal total concentrations of the individual transition metal treatments with those concentrations of transition metals previously reported as toxic in the literature. Table 2 shows the different toxic concentrations of transition metals and their effects reported in the literature for rats/mice and humans. As can be seen, some of the total MICs used here are within or slightly higher than the range of doses reported as lethal to rats/mice. For the case of *E. coli* the total concentration MICs of Co, Cd and Ni at 1 mM (59 ppm), 2 mM (225 ppm) and 2 mM (117 ppm), respectively, meet the above criteria. Meanwhile with *B. subtilis* only MICs of Co and Ni at 2 mM (118 ppm) and 2 mM (117 ppm), respectively, can be considered within the range of the reported non-lethal doses.

**Table 2:** Toxicity data of transition metals.

	Concentration (mg/Kg)	Effect	Reference
Rats/Mice			
Cu	300–794	LD_50_	66, 67
Zn	337–1710	LD_50_	67, 68
Co	42.4	LD_50_	69
Cd	100–300	LD_50_	70
Ni	39–46	LD_50_	71
Human (oral)			
Cu	0.091–6	Gastrointestinal^1^	66
Zn	0.5–6.7	↓Cortisol, ↑LDL, gastrointestinal	68
Co	0.18–1	Gastrointestinal	69
Cd	0.07	Gastrointestinal	70
Ni	7.1–35.7	Gastrointestinal	71
Human (dermal)*			
Cu	2–5% as CuSO_4_ (20 000–50 000)	Irritation	66, 67
Zn	1–2.5% as ZnSO_4_ (10 000–25 000)	May present irritation	67, 68
Co	1% as CoCl_2_ (10 000)	Contact allergy	69
Cd	2% as CdCl_2_ (20 000)	Irritation	70
Ni	0.01–0.4% as NiSO_4_ (100–4 000)	Contact/allergy dermatitis	71
H9C2 cells			
Cu	2	IC_50_	This work
Zn	3	IC_50_	This work
Co	20	IC_50_	This work
Cd	0.1	IC_50_	This work
Ni	33	IC_50_	This work

^1^Gastrointestinal effects may include nausea, vomiting, diarrhea, abdominal cramps. *Dermal exposures expressed the metals salt reported in each case.

However, as previously described in this work, when testing the metals in combinations with Ag, the MICs were reduced by different fractions, depending on the metal and the microorganism. In *E. coli*, the total concentrations used for the combinations were Cu 1 mM (64 ppm), Zn 1 mM (65 ppm), Cd 0.5 mM (56 ppm) and Ni 0.5 mM (59 ppm). In *B. subtilis* these concentrations were Cu 0.5 mM (32 ppm), Zn 0.03 mM (16 ppm), Co 0.25 mM (15 ppm) and Ni 1 mM (59 ppm). Interestingly, the final concentrations used for the combinatorial treatments are all lower than those reported as lethal for mice and rats. However, these concentrations are still higher than those reported as harmful in human oral cases.

To explore the cytotoxic effects of the transition metals and STMCs tested in this work, we first performed viability experiments in human keratinocytes (HaCat cells). Supplementary Figure S4 shows the viability for HaCat cells in culture treated with the transition metals used in this work, at the concentrations reported as the *E. coli* nominal metal MICs. Across the range of concentrations we observed no significant viability (p < 0.05). Most of the metals exhibited an average toxicity of 90%, except Ni with 55% at 24 h of treatment.

**Figure 7:**
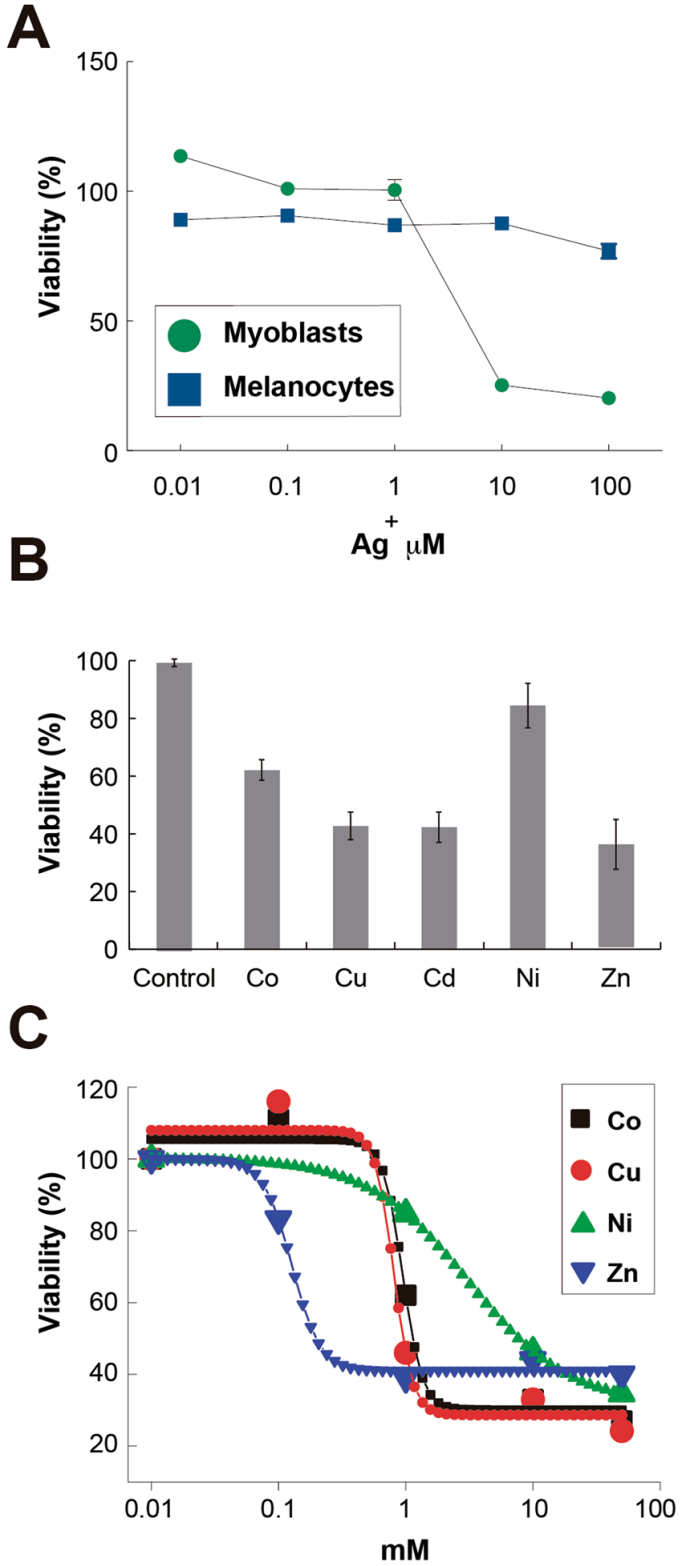
Cytotoxicity effects of Ag and transition metals on H9c2 and B16F10 cells. (**A**) H9c2 and B16F10 cells were treated with Ag at the indicated concentrations for 24 h and viability was determined. (**B**) Effect of transition metals on the viability of cells H9c2. Cells were treated with each transition metal at a concentration of 1 mM for 48 h and viability was determined. Bars represent means of three independent experiments and their respective standard deviations. (**C**) Dose-response effect of transition metals on the viability of H9c2 cells. Cells were treated with transition metals at the stated concentration for 48 h before viability was determined. Indicator points represent means of three independent experiments and their respective standard deviations. Values are mean ± SD (n = 3 experiments at least for each treatment).

Toxicity experiments were performed in another cell line, H9c2 cells, to explore an additional toxicity assay. Figure 7A shows a calculated IC50 value of 3.12 µM for the treatment with individual Ag. This result indicates a strong cytotoxicity of the individual metal. Additional toxicity assays were performed, in H9c2 cells, for the rest of the transition metals, at the nominal concentrations at which they showed antibacterial activity (1 mM). As can be observed in Fig. 7B, Ni and Co did not produce important effects, showing only 15% and 38% cytotoxicity values, respectively. However, Cu and Zn showed about 60% cytotoxicity at the same concentration. Cd, on the other hand, showed to be moderately toxic with about 50% cytotoxicity. Additional concentrations were tested for each of the metals and, with exception of Cd, a typical dose-response effect was observed (Fig. 7C). IC_50_s were calculated and the results show that for all the metals, except Zn and Cd, values are higher (Table 2) than the respective MIC values reported in this work for the transition metals (Table 1). Ni showed to be the less toxic for H9c2 cells, as it needed about 16.5-fold concentration increase in order to reach its IC_50_. Co reaches its IC_50_ with a 20-fold concentration increase. Zn IC_50_ is similar to its MIC value. Due to the similar cytotoxicity of Cd at all tested concentrations, it was not possible to adjust a typical curve to calculate its IC_50_ value, but according to the trend observed, it is lower than 0.1 mM.

Even though the individual metals and STMCs showed cytotoxicity against some of the different cell lines, one explanation could be the difference in the concentrations of free ions available in the medium used in the experiments to calculate MICs (LB), compared to the concentration of free ions in the medium used to calculate the IC_50_ cytotoxic dose (sDMEM and DMEM). Therefore, the free ion concentrations needed to be correlated in both experiments to correctly determine the effect of the different metals and the STMCs. Supplementary Table S7 shows the calculated free ion concentrations corresponding to the nominal concentrations for each of the different media used. As can be observed, some metals, at equivalent nominal concentrations, show differences in the calculated free ion concentrations for the different culture medium. Based on this data, the STMCs for three transition metals were adjusted to perform additional cytotoxicity assays at equivalent free ion concentrations. Table 3 shows the new nominal concentrations used. A comparative of the free ions calculated for these mediums is reported in Supplementary Table S7.

**Table 3:** Correction to metal nominal concentration in DMEM.

Transition metal	Nominal concentration	DMEM free ions*	LB equivalent concentrations
Ag	15 µM	11.4 nM	30 µM
	7.5 µM	5.7 nM	15 µM
	3.75 µM	2.8 nM	7.5 µM
Cu	0.3 mM	2.79 µM	2 mM
	0.08 mM	123 nM	1 mM
Co	0.75 mM	319 µM	0.5 mM
	0.4 mM	170 µM	0.25 mM

*Free ions calculated with VMINTEQ software.

**Figure 8:**
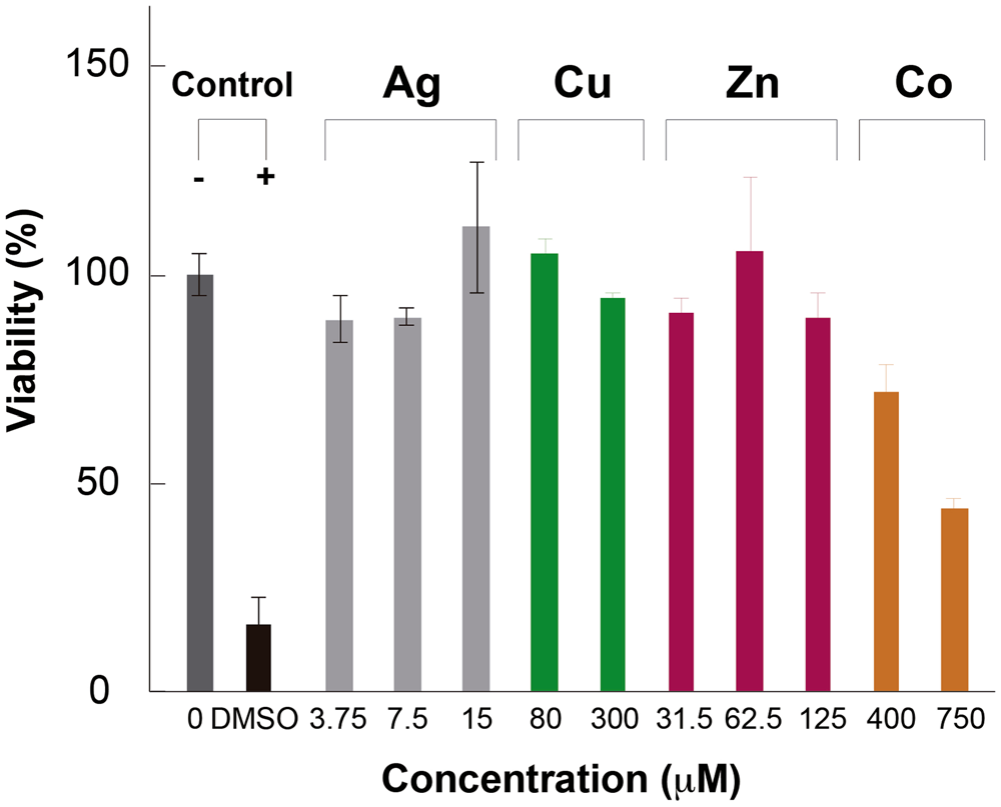
Cytotoxicity of Individual Transition Metals on HaCat cells. Effect of transition metals on the viability of HaCat cells. Cells were treated with individual treatments of transition metals at different concentrations for 24 h and viability was determined. Data are expressed as percent of control mean ± SEM and analyzed by one-way ANOVA, with a Tukey post hoc test. **p < 0.01 vs. control; N = 3.

**Figure 9:**
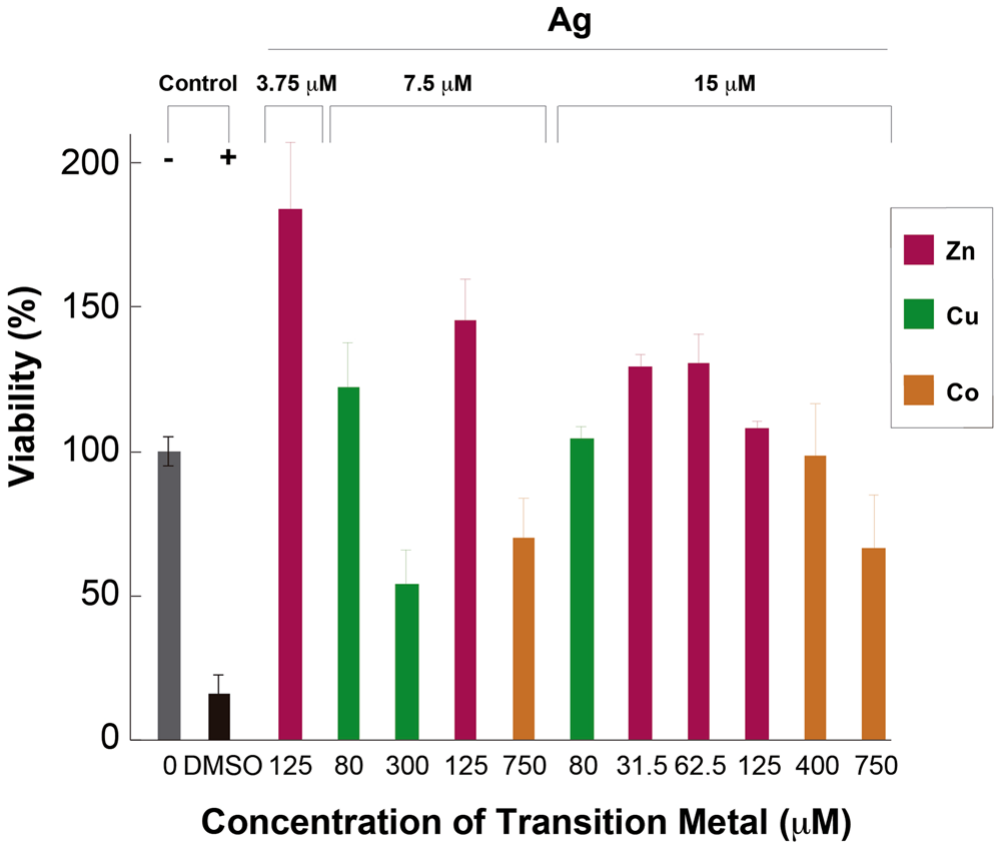
Cytotoxicity of STMCs on HaCat cells. Effect of STMCs on the viability of HaCat cells. Cells were treated with STMCs at different concentrations for 24 h and viability was determined. Data are expressed as percent of control mean ± SEM and analyzed by one-way ANOVA, with a Tukey post hoc test. **p < 0.01 vs. control; N = 3.

Experiments on HaCat cells involving 24 h treatments with transitions metals at the equivalent free metal ions concentrations were performed. It was observed that the percentage of viability significantly increased above 90% for all of the metals (p < 0.01) except for Co at 750 μM where the toxicity was maintained (44.00 ± 1.24%) (Fig. 8). The same phenotype was noticed for the STMCs, all of them showed viabilities above 90%, with the exception of the Ag-Co STMC, with 750 μM Co, which showed a viability of 60%, and the Ag-Cu STMC at the highest concentration of 300 μM Cu, which showed a viability of 50% (Fig. 9). A positive cytotoxicity control treatment with 10% DMSO was used in both experiments, and a viability of 16.32 ± 3.54% was obtained.

As a complementary test to the MTT assay, the cells were evaluated qualitatively with an optical microscope (200X) (Figures S5 and S6). Compared to the control group, notable changes in the monolayer confluence and cell morphology, as well as an increase in the number of floating cells, were markedly seen in groups such as Co 750 μM (Fig. S5F) and also in the positive control (10% DMSO) (Fig. S5B). These results correlate well with the MTT results. On the other hand, groups such as Ag 15μM-Co 750 μM (Fig. S6C) showed a lesser change in monolayer confluence and cell morphology compared to the Co 750 μM treated group (Fig. S5F), but a considerable number of floating cells were observed as compared to control. This was also noted in the MTT assay, where viability of these groups decreased but it was not statistically significant. The rest of the treatments did not seem to suffer changes in the features assessed optically. In an interesting way, some of the groups that showed a higher viability than the control (p < 0.001) in the MTT assay, looked optically similar to the control group. Therefore, such increment can be related more to a higher metabolic activity than to a higher number of cells.

**Figure 10:**
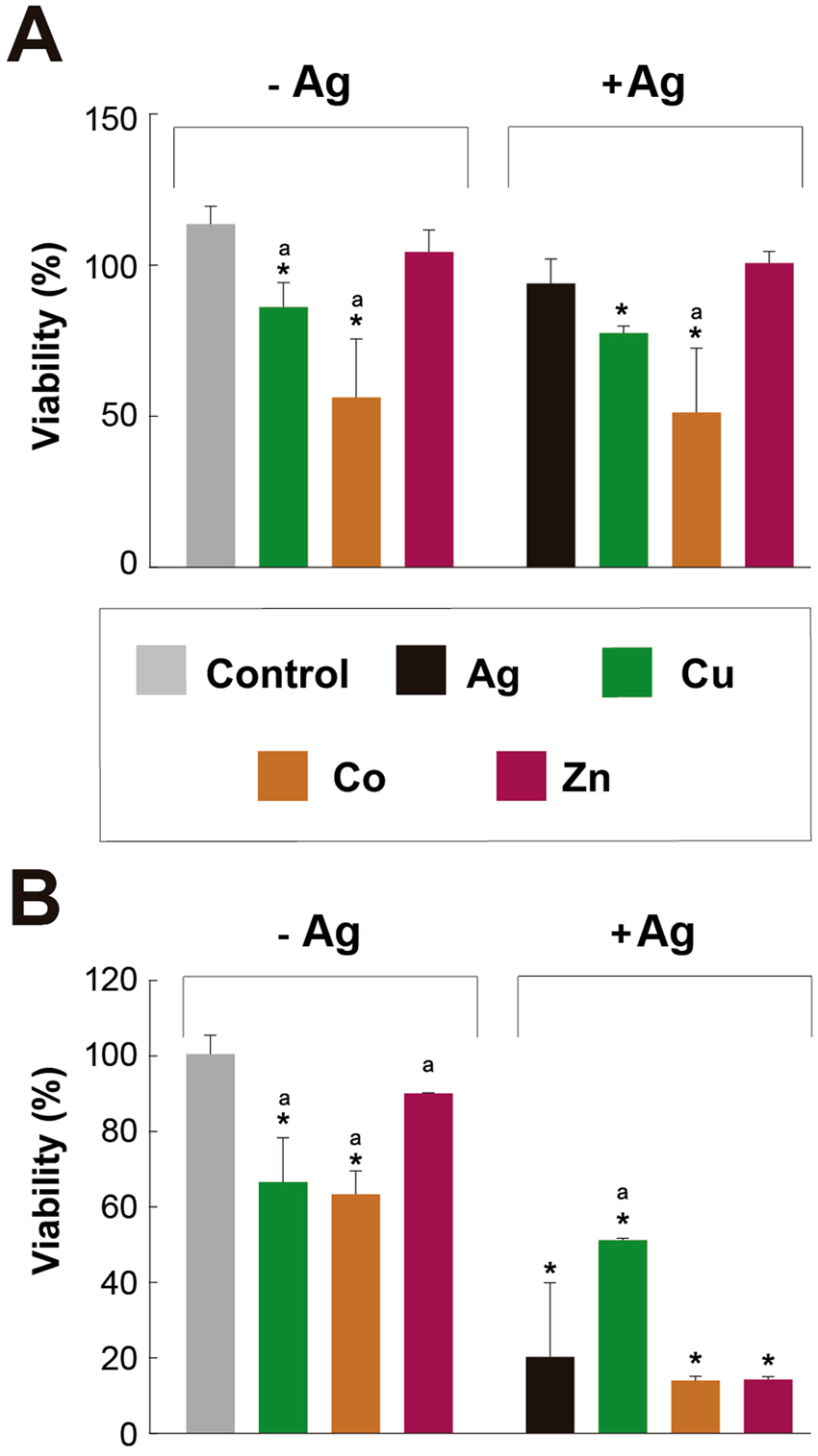
Cytotoxicity effects of Ag and transition metals at concentrations adjusted for speciation on melanocytes and myoblast cells. Effect of transition metals, alone or in combination, with Ag on the viability of (**A**) B16F10 and (**B**) H9c2 cells. Cells were treated with each transition metal (in nominal µM: Cu 80; Co 400; Zn 31.2) alone or in the combination with Ag (15 µM), for 24 h. Values are mean ± SD. *p < 0.05 vs. Control; (a) vs. Ag. (n = 3 experiments at least for each treatment).

As well as with HaCat cells, STMCs at the adjusted free metal ion concentrations were performed in both H9c2 and B16F10 cells. Ag cytotoxicity on H9c2 cells is significantly inhibited when used in combination with Cu (Fig. 10B). When Ag concentration is 15 µM the cytotoxicity is about 50%, but when 80 µM of Cu is added, the cytotoxicity is reduced to about 17%. Combination of Co or Zn does not block Ag cytotoxicity on these cells (Fig. 10B). Cytotoxic effects were also analyzed on melanocytes (B16F10 cells), to observe the effect at the same total concentrations as the experiments with HaCat and H9c2 cells. The results obtained can be compared to those observed in the highly sensitive H9c2 cell line. Ag alone was not found to be cytotoxic to melanocytes (Fig. 7A). In addition, Ag/Zn or Zn alone had not significant cytotoxic effects on B16F10 cells and the combinations of the rest of the metals with Ag did not potentiate their toxicity (Fig. 10A). Taken together, these results suggest that one of the most promising combination is that of Ag with Cu since it shows potential to treat bacterial infections with limited cytotoxic effects on mammalian cells.

The differences in the Ag and STMCs cytotoxic concentrations observed between the different cell lines tested in this work could be explained by the nature of the cells. H9c2 cells are blasts, which normally are sensitive to a variety of insults and they are programmed to die to avoid passing mutations or damage to the progeny. On the other hand, B16F10 and HaCaT cells are skin melanoma and keratinocytes respectively. These cells are more resistant to different conditions as they have mutations in genes that control cell cycle, DNA repair, and apoptosis pathways^72^.

The MIC values obtained for each metal on both Gram-negative and Gram-positive microorganisms and the IC50s obtained in this work, although promising for the development of future therapeutics, cannot be directly compared to their reported LD_50_ values, as our work was made *in vitro* while LD_50_s are calculated from *in vivo* experiments. Even though these *in vitro* studies set the basis for pharmacological applications, as it has been shown in several reports^73, 74^, both bioavailability and biodistribution of the transition metals used here need to be obtained in animal models in order to determine their safety.

Although these concentrations were found to be higher than reported as cytotoxic in human cells and harmful in humans cases by oral administration, an additional important application of these treatments can be found in topical treatment, as seen in Table 2. Skin and soft tissue infections have a great incidence in communities and hospitals^75, 76^ and about 30% of diabetic patients have cutaneous disorders^77^. These kind of infections, like cellulitis, abscesses, diabetic foot infections and surgical site infections caused by multidrug-resistant pathogens, recently have become more common and alternatives to the current treatments are needed^78–80^. Therefore, here we open an area of opportunity for the use of transition metals in dermal treatments. The use of copper as a potential therapeutic in dermal wound healing was reported by Sen *et al*.^81^ noting copper sensitive pathways that regulate wound healing mediators. Some other authors reported the use of transition metals, especially silver, as antimicrobial agents in wound dressing materials and regeneration materials^82–84^. The STMCs reported here can be considered as potential antimicrobial agents in the manufacture of these kind of materials, since we show STMCs to be capable of inhibiting bacterial growth at lower concentrations than each metal as a separate treatment. Furthermore, as shown in this work, the use of STMCs has the advantage of achieving a synergistic effect, allowing the use of lower transition metal doses, and delaying the emergence of resistance to either^85^. Thus, these combinations could be used as a platform to develop novel combinatorial treatments against antibiotic resistance strains. Due to the increments in permeability shown in this work the combinations of metals and silver have potential to be combined with commonly used antibiotics to re-sensitize resistant bacteria. These would allow the decrease in doses of both, antibiotics and transition metals, and at the same time mitigate the possibility of a toxic effect on human cells.

## Conclusions

In correlation with the results provided in this work, we conclude that transition metals, Zn, Cd, Ni and Cu, are able to enhance the inhibitory and bactericidal activities of silver salts against *Escherichia coli* and *B. subtilis*; under the conditions implemented in this study. Nevertheless, the extent to which each STMC affected the behavior of Ag differed significantly. Ag/Ni STMC showed significant potentiation of bactericidal activity when compared to Ag in *E. coli* assays, however no significant effects were observed for treatments in *B. subtilis*. Cu, Ni, and Cd were able to enhance Ag inhibitory activity at 0.25 and 0.5 of their respective MICs, whereas Zn exhibited similar behavior only at 0.5 of its MIC. Meanwhile, Cu, Zn and Co were able to enhance Ag inhibitory activity at these fractions of their respective MICs in *B. subtilis* assays. We demonstrate that STMCs Ag-Cu, Ag-Ni and Ag-Zn exhibit their antimicrobial effect through an increase in cell permeability. The findings described herein provide significant advances in understanding the interactions and effects of combined transition metals and set the basics for future design of metallo-pharmaceutical treatments. In addition, most STMCs tested in this work exhibit antibacterial effects at non-toxic concentrations for human keratinocyte cells. Based on this data, we can conclude that dermic therapeutic applications could be possible, although further investigation need to be taken regarding non-cytotoxic concentrations. Taken together, our results suggest that due to the inherent complexity related to the synergistic effects observed in the systems studied, this work provides a platform for further investigations focused on unveiling deeper correlations to support the use of metallo-pharmaceuticals in future design of therapeutic strategies.

## Electronic supplementary material

Supplementary Information
