# Supplementary material for: Synergistic Antimicrobial Effects of Silver/Transition-metal Combinatorial Treatments
Source: Sci Rep. 2017 Apr 18;7:903. doi: 10.1038/s41598-017-01017-7 (PMC5429853; doi:10.1038/s41598-017-01017-7)
Supplement: Supplementary file 1 — Supplementary Information [file 41598_2017_1017_MOESM1_ESM.pdf]

Synergistic Antimicrobial Effects of Silver/Transition-metal Combinatorial Treatments

*Javier A. Garza-Cervantes[1,2], Arturo Chávez-Reyes[3], Elena C. Castillo[4,5], Gerardo García-Rivas[4,5], Oscar Antonio Ortega-Rivera[6], Eva Salinas[6], Margarita Ortiz-Martínez[1], Sara Leticia Gómez-Flores[1], Jorge A. Peña-Martínez[1], Alan Pepi-Molina[7], Mario T. Treviño-González[8], Xristo Zarate[1,2], María Elena Cantú-Cárdenas[1,2], Carlos Enrique Escarcega-Gonzalez[1,2], and J. Rubén Morones-Ramírez[1,2]**

1 Universidad Autónoma de Nuevo León, UANL. Facultad de Ciencias Químicas. Av. Universidad s/n, Cd. Universitaria, 66451, San Nicolás de los Garza, NL, México.
2 Centro de Investigacion en Biotecnologia y Nanotoxicologia, Facultad de Ciencias Quimicas, Universidad Autonoma de Nuevo Leon. Parque de Investigacion e Innovacion Tecnologica, Km. 10 autopista al Aeropuerto Internacional Mariano Escobedo, Apodaca, Nuevo Leon. 66629
3 Centro de Investigación y de Estudios Avanzados del IPN, Unidad Monterrey, Parque PIIT. 66600. Apodaca, Nuevo León, México.
4 Cátedra de Cardiología y Medicina Vascular. Escuela de Medicina. Tecnologico de Monterrey, Monterrey, Nuevo León, México
5 Centro de Investigación Biomédica. Hospital Zambrano-Hellion. Tecnologico de Monterrey, San Pedro Garza-García, Nuevo León, México
6 Departamento de Microbiología, Centro de Ciencias Básicas, Universidad Autónoma de Aguascalientes. Av. Universidad 940, Colonia Ciudad Universitaria C.P. 20131, Aguascalientes, Aguascalientes, Mexico
7 Universidad Autónoma de Nuevo León, UANL. Facultad de Ciencias Biológicas. Av. Universidad s/n, Cd. Universitaria, 66451, San Nicolás de los Garza, NL, México.
8 Universidad Autónoma de Nuevo León, UANL. Facultad de Ingeniería Mecánica y Eléctrica. Av. Universidad s/n, Cd. Universitaria, 66451, San Nicolás de los Garza, NL, México.

[*] Corresponding Author:

Facultad de Ciencias Químicas, Universidad Autónoma de Nuevo León, Pedro de Alba, S/N, San Nicolás de los Garza, Nuevo León, México.

E-mail: jose.moronesrmr@uanl.edu.mx, morones.ruben@gmail.com

Tel: +52-1-818-329-4000 Ext. 6295

Supplementary Information

MIC determination through Checkerboard assays for the STMC´s

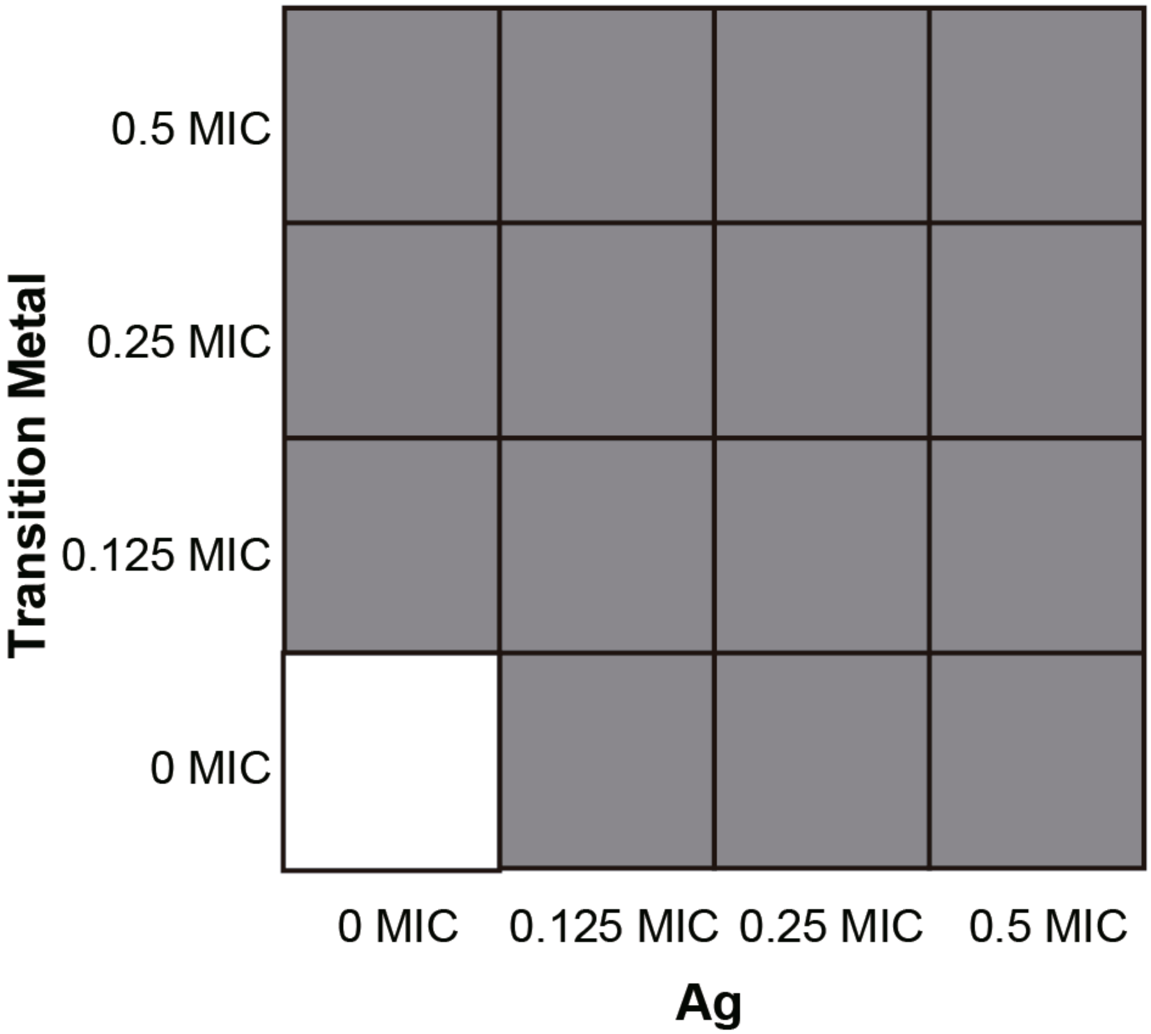

Figure S1. Checkerboard test layout. Component X (Silver) and component Y (Transition metal) combination, the *white well* corresponds to the control culture and all the *gray wells* include treated cultures, either individual or combinatorial treatments.

Table S1. LB medium components and metal species at *Escherichia coli* MICs

Component	Concentration* in LB (M)	Components concentration* (M) in LB using: Ag 60 µM	Cu 4 mM	Zn 2 mM	Cd 2 mM	Ni 2 mM	Co 1 mM
**Ca^{2+}**	$6.40x10^{-5}$	$6.40x10^{-5}$	$6.24x10^{-5}$	$6.28x10^{-5}$	$6.26x10^{-5}$	$6.29x10^{-5}$	$6.42x10^{-5}$
**Fe^{2+}**	$4.51x10^{-6}$	$4.51x10^{-6}$	$4.60x10^{-6}$	$4.60x10^{-6}$	$4.52x10^{-6}$	$4.50x10^{-6}$	$4.58x10^{-6}$
**Mg^{2+}**	$1.78x10^{-4}$	$1.78x10^{-4}$	$1.72x10^{-4}$	$1.75x10^{-4}$	$1.75x10^{-4}$	$1.75x10^{-4}$	$1.78x10^{-4}$
**K^{+}**	$4.70x10^{-3}$	$4.70x10^{-3}$	$4.66x10^{-3}$	$4.68x10^{-3}$	$4.68x10^{-3}$	$4.68x10^{-3}$	$4.69x10^{-3}$
**Na^{+}**	$1.78x10^{-1}$	$1.78x10^{-1}$	$1.78x10^{-1}$	$1.78x10^{-1}$	$1.78x10^{-1}$	$1.78x10^{-1}$	$1.78x10^{-1}$
**Cl^{-}**	$1.65x10^{-1}$	$1.65x10^{-1}$	$1.65x10^{-1}$	$1.65x10^{-1}$	$1.63x10^{-1}$	$1.65x10^{-1}$	$1.67x10^{-1}$
**HPO_4^{2-}**	$1.41x10^{-3}$	$1.41x10^{-3}$	$1.31x10^{-3}$	$1.31x10^{-3}$	$1.35x10^{-3}$	$1.39x10^{-3}$	$1.37x10^{-3}$
**$H_2PO_4^{-}$**	$1.39x10^{-3}$	$1.39x10^{-3}$	$1.29x10^{-3}$	$1.30x10^{-3}$	$1.34x10^{-3}$	$1.37x10^{-3}$	$1.35x10^{-3}$
**H-Glutamate^{-}**	$3.22x10^{-3}$	$3.22x10^{-3}$	$3.76x10^{-5}$	$2.86x10^{-3}$	$3.21x10^{-3}$	$2.12x10^{-3}$	$3.06x10^{-3}$
**Glutamate^{2-}**	$1.00x10^{-5}$	$1.00x10^{-5}$	$1.18x10^{-7}$	$8.93x10^{-6}$	$1.00x10^{-5}$	$6.63x10^{-6}$	$9.54x10^{-6}$
**Glycine^{-}**	$2.76x10^{-6}$	$2.76x10^{-6}$	$5.09x10^{-8}$	$2.33x10^{-6}$	$2.74x10^{-6}$	$1.76x10^{-6}$	$2.61x10^{-6}$

Major Ag species (M)		Major Cu species (M)		Major Zn species (M)		Major Cd species (M)		Major Ni species (M)		Major Co species (M)	
$AgCl^{2-}$	$4.77x10^{-5}$	Cu-Glutamate $^{(aq)}$	$1.98x10^{-3}$	Zn^{2+}	$9.80x10^{-4}$	$CdCl^{+}$	$1.14x10^{-3}$	Ni-Glutamate $_{(aq)}$	$9.70x10^{-4}$	Co^{2+}	$6.56x10^{-4}$
$AgCl_3^{2-}$	$8.91x10^{-6}$	Cu-Glycine^{+}	$6.30x10^{-4}$	Zn-Glutamate $_{(aq)}$	$3.32x10^{-4}$	$CdCl_2$ $_{(aq)}$	$4.27x10^{-4}$	Ni^{2+}	$5.32x10^{-4}$	Co-Glutamate $_{(aq)}$	$1.54x10^{-4}$
AgCl $_{(aq)}$	$3.40x10^{-6}$	Cu-(Glutamate)$_2^{2-}$	$6.09x10^{-4}$	$ZnHPO_4$ $_{(aq)}$	$3.06x10^{-4}$	Cd^{2+}	$2.29x10^{-4}$	Ni-Glycine^{+}	$3.07x10^{-4}$	$CoHPO_4$ $_{(aq)}$	$1.18x10^{-4}$
Ag^{+}	$2.28x10^{-8}$	$CuHPO_4$ $_{(aq)}$	$3.28x10^{-4}$	$ZnCl^{+}$	$1.56x10^{-4}$	$CdHPO_4$ $_{(aq)}$	$1.81x10^{-4}$	$NiHPO_4$ $_{(aq)}$	$7.65x10^{-5}$	Co-Glycine^{+}	$5.16x10^{-5}$
Ag-Glutamate^{+}	$1.18x10^{-9}$	Cu^{2+}	$1.64x10^{-4}$	Zn-Glycine^{+}	$1.42x10^{-4}$	Cd-Glutamate $_{(aq)}$	$9.81x10^{-6}$	Ni-(Glutamate)$_2^{2-}$	$6.66x10^{-5}$	$CoCl^{+}$	$1.56x10^{-5}$

*Concentration values calculated with VMinteq Software

Table S1. LB medium components and metal species at *Escherichia coli* MICs

Table S2. LB medium components and metal species at Bacillus subtilis MICs

Component	Concentration* in LB (M)	Components concentration* (M) in LB using: Ag 60 μM	Cu 2 mM	Zn 0.25 mM	Cd 0.015 mM	Ni 2 mM	Co 1 mM
**Ca^{2+}**	$6.40x10^{-5}$	$6.40x10^{-5}$	$6.36x10^{-5}$	$6.38x10^{-5}$	$6.40x10^{-5}$	$6.29x10^{-5}$	$6.42x10^{-5}$
**Fe^{2+}**	$4.51x10^{-6}$	$4.51x10^{-6}$	$4.51x10^{-6}$	$4.52x10^{-6}$	$4.51x10^{-6}$	$4.50x10^{-6}$	$4.58x10^{-6}$
**Mg^{2+}**	$1.78x10^{-4}$	$1.78x10^{-4}$	$1.75x10^{-4}$	$1.77x10^{-4}$	$1.78x10^{-4}$	$1.75x10^{-4}$	$1.78x10^{-4}$
**K^{+}**	$4.70x10^{-3}$	$4.70x10^{-3}$	$4.68x10^{-3}$	$4.69x10^{-3}$	$4.70x10^{-3}$	$4.68x10^{-3}$	$4.69x10^{-3}$
**Na^{+}**	$1.78x10^{-1}$	$1.78x10^{-1}$	$1.78x10^{-1}$	$1.78x10^{-1}$	$1.78x10^{-1}$	$1.78x10^{-1}$	$1.78x10^{-1}$
**Cl^{-}**	$1.65x10^{-1}$	$1.65x10^{-1}$	$1.65x10^{-1}$	$1.65x10^{-1}$	$1.65x10^{-1}$	$1.65x10^{-1}$	$1.67x10^{-1}$
**HPO_4^{2-}**	$1.41x10^{-3}$	$1.41x10^{-3}$	$1.41x10^{-3}$	$1.39x10^{-3}$	$1.41x10^{-3}$	$1.39x10^{-3}$	$1.37x10^{-3}$
**$H_2PO_4^{-}$**	$1.39x10^{-3}$	$1.39x10^{-3}$	$1.39x10^{-3}$	$1.38x10^{-3}$	$1.39x10^{-3}$	$1.37x10^{-3}$	$1.35x10^{-3}$
**H-Glutamate^{-}**	$3.22x10^{-3}$	$3.22x10^{-3}$	$4.41x10^{-4}$	$3.17x10^{-3}$	$3.22x10^{-3}$	$2.12x10^{-3}$	$3.06x10^{-3}$
**Glutamate^{2-}**	$1.00x10^{-5}$	$1.00x10^{-5}$	$1.38x10^{-6}$	$9.87x10^{-6}$	$1.00x10^{-5}$	$6.63x10^{-6}$	$9.54x10^{-6}$
**Glycine^{-}**	$2.76x10^{-6}$	$2.76x10^{-6}$	$6.10x10^{-7}$	$2.70x10^{-6}$	$2.76x10^{-6}$	$1.76x10^{-6}$	$2.61x10^{-6}$

Major Ag species (M)		Major Cu species (M)		Major Zn species (M)		Major Cd species (M)		Major Ni species (M)		Major Co species (M)	
$AgCl^{2-}$	$4.77x10^{-5}$	Cu-(Glutamate)$_2^{2-}$	$1.23x10^{-3}$	Zn^{2+}	$1.19x10^{-4}$	$CdCl^{+}$	$8.51x10^{-7}$	Ni-Glutamate $_{(aq)}$	$9.70x10^{-4}$	Co^{2+}	$6.56x10^{-4}$
$AgCl_3^{2-}$	$8.91x10^{-6}$	Cu-Glutamate $_{(aq)}$	$3.40x10^{-4}$	Zn-Glutamate $_{(aq)}$	$4.49x10^{-5}$	$CdCl_2$ $_{(aq)}$	$3.25x10^{-7}$	Ni^{2+}	$5.32x10^{-4}$	Co-Glutamate $_{(aq)}$	$1.54x10^{-4}$
AgCl $_{(aq)}$	$3.40x10^{-6}$	Cu-(Glycine)$_2$ $_{(aq)}$	$3.13x10^{-4}$	$ZnHPO_4$ $_{(aq)}$	$3.97x10^{-5}$	Cd^{2+}	$1.69x10^{-7}$	Ni-Glycine^{+}	$3.07x10^{-4}$	$CoHPO_4$ $_{(aq)}$	$1.18x10^{-4}$
Ag^{+}	$2.28x10^{-8}$	Cu-Glycine^{+}	$1.11x10^{-4}$	Zn-Glycine^{+}	$2.01x10^{-5}$	$CdHPO_4$ $_{(aq)}$	$1.40x10^{-7}$	$NiHPO_4$ $_{(aq)}$	$7.65x10^{-5}$	Co-Glycine^{+}	$5.16x10^{-5}$
Ag-Glutamate^{+}	$1.18x10^{-9}$	Cu^{2+}	$2.39x10^{-6}$	$ZnCl^{+}$	$1.90x10^{-5}$	Cd-Glutamate $_{(aq)}$	$7.31x10^{-9}$	Ni-(Glutamate)$_2^{2-}$	$6.66x10^{-5}$	$CoCl^{+}$	$1.56x10^{-5}$

*Concentration values calculated with VMinteq Software

Table S2. LB medium components and metal species at Bacillus subtilis MICs

Calculations of Free Ions in Each of the Media Tested

Table S3. Metals free ions concentrations (M) at *E. coli* checkerboard combinations.

	Ag/Cu (μM/mM)								
	30/2	30/1	30/0.5	15/2	15/1	15/0.5	7.5/2	7.5/1	7.5/0.5
**Cu^{2+}**	2.39x10^{-6}	1.14x10^{-7}	2.80x10^{-8}	2.39x10^{-6}	1.14x10^{-7}	2.80x10^{-8}	2.39x10^{-6}	1.14x10^{-7}	2.80x10^{-8}
**Ag^{+}**	1.14x10^{-8}	1.14x10^{-8}	1.14x10^{-8}	5.71x10^{-9}	5.71x10^{-9}	5.70x10^{-9}	2.86x10^{-9}	2.85x10^{-9}	2.85x10^{-9}
	Ag/Zn (μM/mM)								
	30/1	30/0.5	30/0.25	15/1	15/0.5	15/0.25	7.5/1	7.5/0.5	7.5/0.25
**Zn^{2+}**	4.82x10^{-4}	2.39x10^{-4}	1.19x10^{-4}	4.82x10^{-4}	2.39x10^{-4}	1.19x10^{-4}	4.82x10^{-4}	2.39x10^{-4}	1.19x10^{-4}
**Ag^{+}**	1.14x10^{-8}	1.14x10^{-8}	1.14x10^{-8}	5.71x10^{-9}	5.71x10^{-9}	5.70x10^{-9}	2.86x10^{-9}	2.85x10^{-9}	2.85x10^{-9}
	Ag/Cd (μM/mM)								
	30/1	30/0.5	30/0.25	15/1	15/0.5	15/0.25	7.5/1	7.5/0.5	7.5/0.25
**Cd^{2+}**	1.13x10^{-4}	5.64x10^{-5}	2.82x10^{-5}	1.13x10^{-4}	5.64x10^{-5}	2.82x10^{-5}	1.13x10^{-4}	5.64x10^{-5}	2.82x10^{-5}
**Ag^{+}**	1.13x10^{-8}	1.13x10^{-8}	1.14x10^{-8}	5.63x10^{-9}	5.67x10^{-9}	5.68x10^{-9}	2.82x10^{-9}	2.83x10^{-9}	2.84x10^{-9}
	Ag/Co (μM/mM)								
	30/0.5	30/0.25	30/0.125	15/0.5	15/0.25	15/0.125	7.5/0.5	7.5/0.25	7.5/0.125
**Co^{2+}**	3.26x10^{-4}	1.62x10^{-4}	8.09x10^{-5}	3.26x10^{-4}	1.62x10^{-4}	8.09x10^{-5}	3.26x10^{-4}	1.62x10^{-4}	8.09x10^{-5}
**Ag^{+}**	1.13x10^{-8}	1.13x10^{-8}	1.14x10^{-8}	5.64x10^{-9}	5.67x10^{-9}	5.68x10^{-9}	2.82x10^{-9}	2.83x10^{-9}	2.84x10^{-9}
	Ag/Ni (μM/mM)								
	30/1	30/0.5	30/0.25	15/1	15/0.5	15/0.25	7.5/1	7.5/0.5	7.5/0.25
**Ni^{2+}**	2.26x10^{-4}	1.04x10^{-4}	4.97x10^{-5}	2.26x10^{-4}	1.04x10^{-4}	4.97x10^{-5}	2.26x10^{-4}	1.04x10^{-4}	4.97x10^{-5}
**Ag^{+}**	1.14x10^{-8}	1.14x10^{-8}	1.14x10^{-8}	5.71x10^{-9}	5.70x10^{-9}	5.70x10^{-9}	2.85x10^{-9}	2.85x10^{-9}	2.85x10^{-9}

***Free ions calculated with VMinteq Software**

Table S3. Metals free ions concentrations (M) at *E. coli* checkerboard combinations.

Table S4. Metals free ions concentrations (M) at B. subtilis checkerboard combinations.

	Ag/Cu (µM/mM)								
	30/1	30/0.5	30/0.25	15/1	15/0.5	15/0.25	7.5/1	7.5/0.5	7.5/0.25
**Cu^{2+}**	$1.14x10^{-7}$	$2.80x10^{-8}$	$1.04x10^{-8}$	$1.14x10^{-7}$	$2.80x10^{-8}$	$1.04x10^{-8}$	$1.14x10^{-7}$	$2.80x10^{-8}$	$1.04x10^{-8}$
**Ag^{+}**	$1.14x10^{-8}$	$1.14x10^{-8}$	$1.14x10^{-8}$	$5.71x10^{-9}$	$5.70x10^{-9}$	$5.70x10^{-9}$	$2.85x10^{-9}$	$2.85x10^{-9}$	$2.85x10^{-9}$

	Ag/Zn (µM/mM)								
	30/0.125	30/0.0625	30/0.03125	15/0.125	15/0.0625	15/0.03125	7.5/0.125	7.5/0.0625	7.5/0.03125
**Zn^{2+}**	$5.92x10^{-5}$	$2.96x10^{-5}$	$1.48x10^{-5}$	$5.92x10^{-5}$	$2.96x10^{-5}$	$1.48x10^{-5}$	$5.92x10^{-5}$	$2.96x10^{-5}$	$1.48x10^{-5}$
**Ag^{+}**	$1.14x10^{-8}$	$1.14x10^{-8}$	$1.14x10^{-8}$	$5.70x10^{-9}$	$5.70x10^{-9}$	$5.70x10^{-9}$	$2.85x10^{-9}$	$2.85x10^{-9}$	$2.85x10^{-9}$

	Ag/Cd (µM/mM)								
	30/0.0075	30/0.00375	30/0.001875	15/0.0075	15/0.00375	15/0.001875	7.5/0.0075	7.5/0.00375	7.5/0.001875
**Cd^{2+}**	$8.47x10^{-7}$	$4.24x10^{-7}$	$2.12x10^{-7}$	$8.47x10^{-7}$	$4.24x10^{-7}$	$2.12x10^{-7}$	$8.47x10^{-7}$	$4.23x10^{-7}$	$2.12x10^{-7}$
**Ag^{+}**	$1.14x10^{-8}$	$1.14x10^{-8}$	$1.14x10^{-8}$	$5.70x10^{-9}$	$5.70x10^{-9}$	$5.70x10^{-9}$	$2.85x10^{-9}$	$2.85x10^{-9}$	$2.85x10^{-9}$

	Ag/Co (µM/mM)								
	30/0.5	30/0.25	30/0.125	15/0.5	15/0.25	15/0.125	7.5/0.5	7.5/0.25	7.5/0.125
**Co^{2+}**	$3.26x10^{-4}$	$1.62x10^{-4}$	$8.09x10^{-5}$	$3.26x10^{-4}$	$1.62x10^{-4}$	$8.09x10^{-5}$	$3.26x10^{-4}$	$1.62x10^{-4}$	$8.09x10^{-5}$
**Ag^{+}**	$1.13x10^{-8}$	$1.13x10^{-8}$	$1.14x10^{-8}$	$5.64x10^{-9}$	$5.67x10^{-9}$	$5.68x10^{-9}$	$2.82x10^{-9}$	$2.83x10^{-9}$	$2.84x10^{-9}$

	Ag/Ni (µM/mM)								
	30/1	30/0.5	30/0.25	15/1	15/0.5	15/0.25	7.5/1	7.5/0.5	7.5/0.25
**Ni^{2+}**	$2.26x10^{-4}$	$1.04x10^{-4}$	$4.97x10^{-5}$	$2.26x10^{-4}$	$1.04x10^{-4}$	$4.97x10^{-5}$	$2.26x10^{-4}$	$1.04x10^{-4}$	$4.97x10^{-5}$
**Ag^{+}**	$1.14x10^{-8}$	$1.14x10^{-8}$	$1.14x10^{-8}$	$5.71x10^{-9}$	$5.70x10^{-9}$	$5.70x10^{-9}$	$2.85x10^{-9}$	$2.85x10^{-9}$	$2.85x10^{-9}$

***Free ions calculated with VMinteq software**

Table S4. Metals free ions concentrations (M) at B. subtilis checkerboard combinations.

Sub inhibitory STMCs effect on and B. subtilis

After the combination performed on checkerboard assays, extra dilutions were assessed. We observed that for both strains no significant inhibitory effect was achieved. We also observed that with Ag-Ni and Ag-Co treatments, *B. subtilis* wells exhibit a hormesis effect from the combinations made as for an isobologram analysis.

Ag µM\Ni mM	0.5	0.25	0.125	0.0625	0.03125	0.015625	0.0078125
15			136.40				
7.5			132.61	132.13			
3.75	173.74	155.31	142.51	122.91	118.74		
1.875		157.83	139.11	133.58	124.85	117.68	
0.9375			143.87	132.71	129.22	115.54	91.29
0.46875				122.43	125.05	120.88	94.98
0.234375					108.27	97.50	100.70

Table S5. Effect in *B. subtilis* growth caused by Ag-Ni combinations. Growth percent of *B. subtilis* when exposed at lower concentrations than checkerboard assays. Ag^{+} and Ni^{2+} nominal concentrations expressed in µM and mM, respectively.

Ag µM\Co mM	0.25	0.125	0.0625	0.03125	0.015625	0.0078125	0.00390625
15			133.12				
7.5			136.72	123.17			
3.75	129.94	126.77	129.17	128.14	124.37		
1.875		124.88	129.43	129.34	131.57	114.84	
0.9375			121.02	125.31	134.15	114.93	99.23
0.46875				109.61	117.50	119.73	103.17
0.234375					102.83	105.66	106.78

Table S6. Effect in *B. subtilis* growth caused by Ag-Co combinations. Growth percent of *B. subtilis* when exposed at lower concentrations than checkerboard assays. Ag^{+} and Co^{2+} nominal concentrations expressed in µM and mM, respectively.

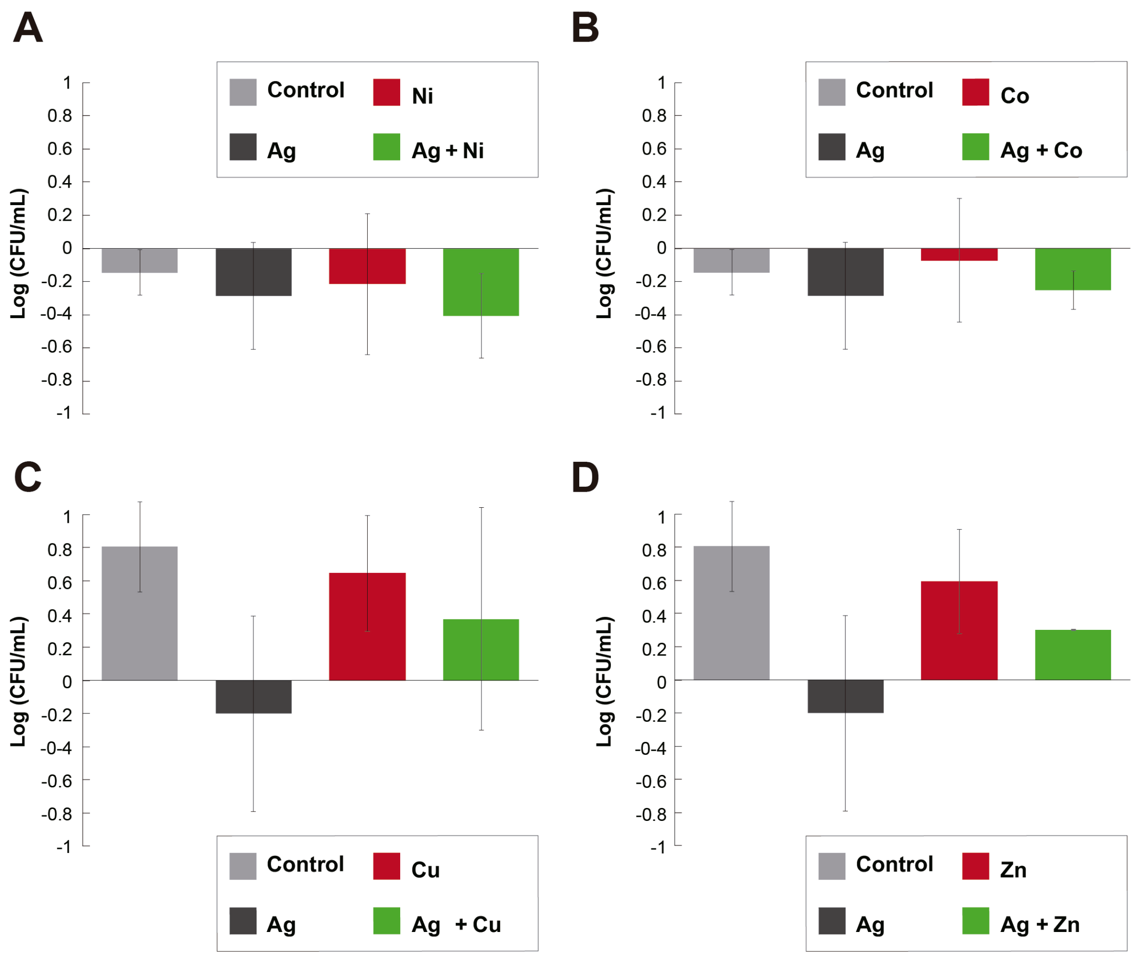

Figure S2. Bactericidal Effect of Ag^+ Potentiated by Transition Metals. Log change in CFUs/mL with respect to time zero, in *Bacillus subtilis* ATCC 23857 after 1 hour treatment with: LB (control), Ag, specific transition metal and their combination: (A) Ag 30 µM, Ni 0.5 mM and the combination; (B) Ag 30 µM, Cd 1 mM and the combination; (C) Ag 15 µM, Cu 1 mM and 2 mM, and the respective combinations; (D) Ag 30 µM, Zn 0.5 mM and the combination. *** Corresponds to a P<0.05, tested with an ANOVA, that there is a difference with respect to the control and each of the individual treatments. Error bars correspond to the standard deviation from experiments performed in triplicates.

Flow cytometry analysis

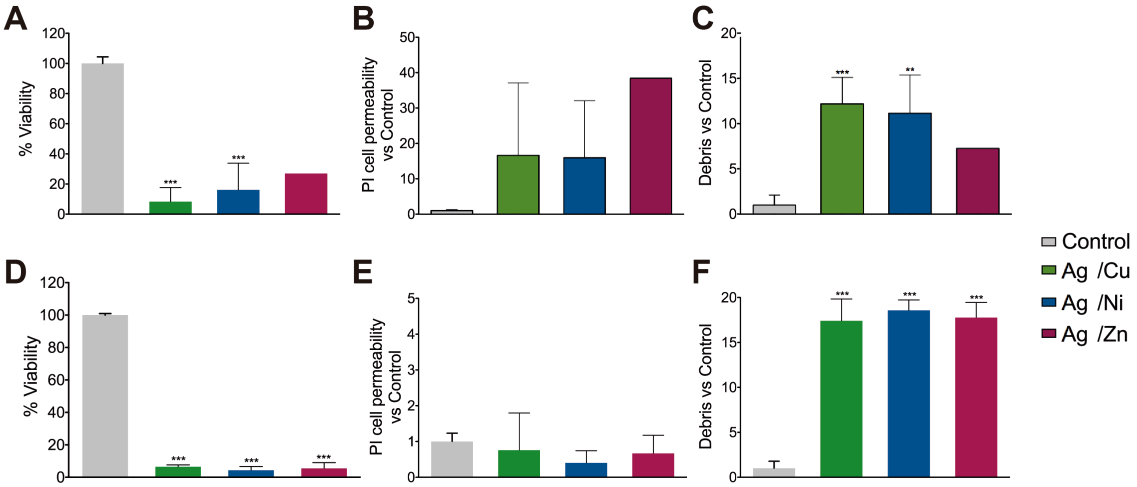

Figure S3

Figure S3. Viability and cell permeability assay of *B. subtilis* and *E. coli*. Bacteria treated with Ag-Cu Ag-Ni and Ag-Zn (30 µM-1mM) by 24h (37 °C). SyBR-G positive cells are represented as % of Viability whereas cell death as PI cell permeability. SyBR-G and PI negative cells were considered cell debris. Values represent mean ± SED (n = 3 experiments for each treatment, except Ag-Zn treatment n=2). $^{**}P < 0.01$ *vs*. Control. $^{***}P < 0.001$ *vs*. Control.

Transition Metal Toxicity on HaCat Cells

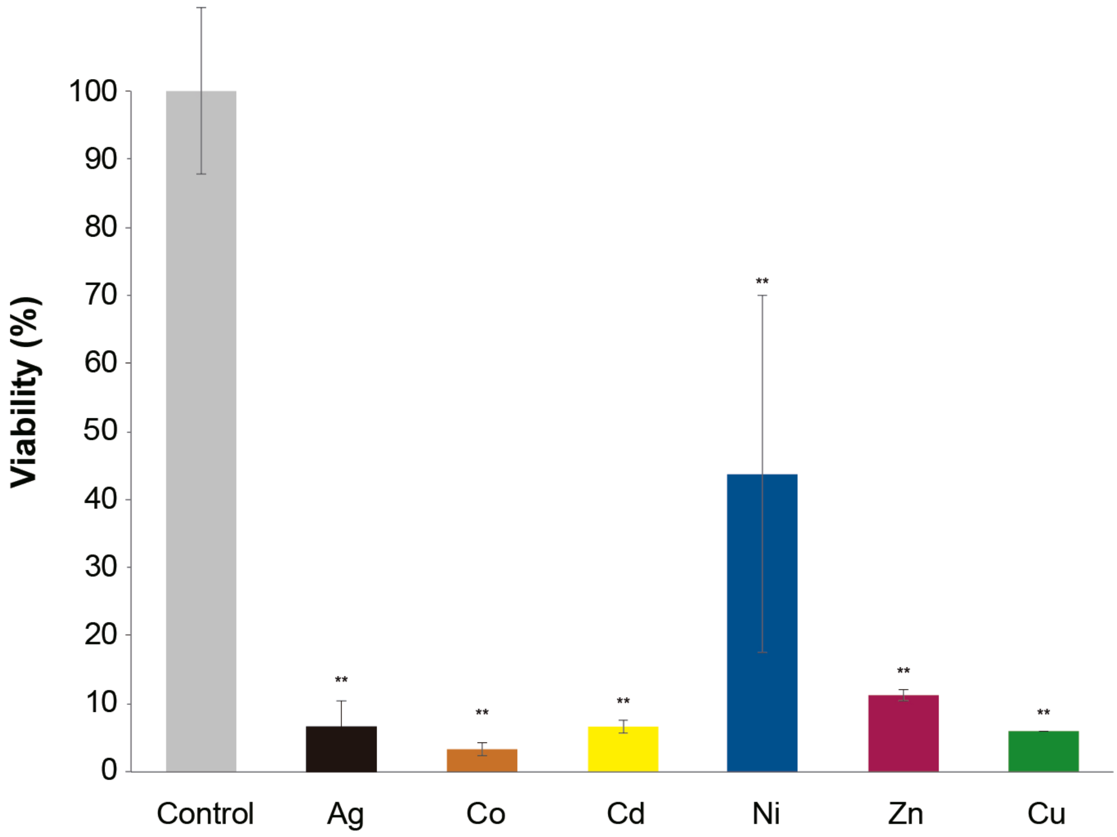

Figure S4

Figure S4. Cytotoxicity of Ag^+ and transition metals on a HaCat cell line. Cells were treated with each transition metal at a concentration of *E. coli* MIC for 24 h and viability was determined. Bars represent means of three independent experiments and their respective standard deviations. ** Corresponds to a $p<0.05$ that there is a difference with respect to the control.

Free Ion Calculation in the Different Media Used

Table S7. Metal free ions* comparison in different culture medium.

Total concentrations		Calculated free ions (M) in LB Medium	sDMEM	DMEM	DMEM/F12
**Ag (µM)**	60	$2.28x10^{-8}$	$4.61x10^{-8}$	$4.60x10^{-8}$	$4.02x10^{-8}$
	30	$1.14x10^{-8}$	$2.30x10^{-8}$	$2.30x10^{-8}$	$2.01x10^{-8}$
	15	$5.70x10^{-9}$	$1.15x10^{-8}$	$1.15x10^{-8}$	$1.00x10^{-8}$
	7.5	$2.85x10^{-9}$	$5.75x10^{-9}$	$5.74x10^{-9}$	$5.02x10^{-9}$
**Cu (mM)**	4	$1.64x10^{-4}$	$1.09x10^{-4}$	$1.09x10^{-4}$	$2.61x10^{-4}$
	2	$2.39x10^{-6}$	$4.88x10^{-5}$	$4.88x10^{-5}$	$1.33x10^{-4}$
	1	$1.14x10^{-7}$	$2.05x10^{-5}$	$2.05x10^{-5}$	$5.86x10^{-5}$
	0.5	$2.80x10^{-8}$	$7.38x10^{-6}$	$7.38x10^{-6}$	$2.15x10^{-5}$
	0.25	$1.04x10^{-8}$	$1.84x10^{-6}$	$1.84x10^{-6}$	$5.50x10^{-6}$
**Zn(mM)**	2	$9.80x10^{-4}$	$9.54x10^{-4}$	$9.54x10^{-4}$	$1.25x10^{-3}$
	1	$4.82x10^{-4}$	$4.75x10^{-4}$	$4.75x10^{-4}$	$6.24x10^{-4}$
	0.5	$2.39x10^{-4}$	$2.37x10^{-4}$	$2.37x10^{-4}$	$3.12x10^{-4}$
	0.25	$1.19x10^{-4}$	$1.18x10^{-4}$	$1.18x10^{-4}$	$1.56x10^{-4}$
	0.125	$5.92x10^{-5}$	$5.91x10^{-5}$	$5.91x10^{-5}$	$7.85x10^{-5}$
	0.0625	$2.96x10^{-5}$	$2.96x10^{-5}$	$2.95x10^{-5}$	$3.97x10^{-5}$
	0.0375	$1.48x10^{-5}$	$1.48x10^{-5}$	$1.48x10^{-5}$	$2.03x10^{-5}$
**Cd (mM)**	2	$2.29x10^{-4}$	$3.11x10^{-4}$	$3.11x10^{-4}$	$3.03x10^{-4}$
	1	$1.14x10^{-4}$	$1.55x10^{-4}$	$1.55x10^{-4}$	$1.50x10^{-4}$
	0.5	$5.67x10^{-5}$	$7.71x10^{-5}$	$7.70x10^{-5}$	$7.48x10^{-5}$
	0.25	$2.83x10^{-5}$	$3.85x10^{-5}$	$3.85x10^{-5}$	$3.73x10^{-5}$
**Co (mM)**	1	$6.56x10^{-4}$	$4.28x10^{-4}$	$4.28x10^{-4}$	$6.55x10^{-4}$
	0.5	$3.26x10^{-4}$	$2.13x10^{-4}$	$2.13x10^{-4}$	$3.26x10^{-4}$
	0.25	$1.62x10^{-4}$	$1.06x10^{-4}$	$1.06x10^{-4}$	$1.62x10^{-4}$
	0.125	$8.09x10^{-5}$	$5.30x10^{-5}$	$5.30x10^{-5}$	$8.11x10^{-5}$
**Ni (mM)**	2	$5.32x10^{-4}$	$6.01x10^{-4}$	$6.01x10^{-4}$	$1.05x10^{-3}$
	1	$2.26x10^{-4}$	$2.89x10^{-4}$	$2.89x10^{-4}$	$5.00x10^{-4}$
	0.5	$1.04x10^{-4}$	$1.41x10^{-4}$	$1.41x10^{-4}$	$2.40x10^{-4}$
	0.25	$4.97x10^{-5}$	$6.91x10^{-5}$	$6.90x10^{-5}$	$1.17x10^{-4}$

***Free ions calculated with VMinteq software**

Table S7. Metal free ions* comparison in different culture medium.

Morphology changes caused by STMCs in human keratinocyte cells

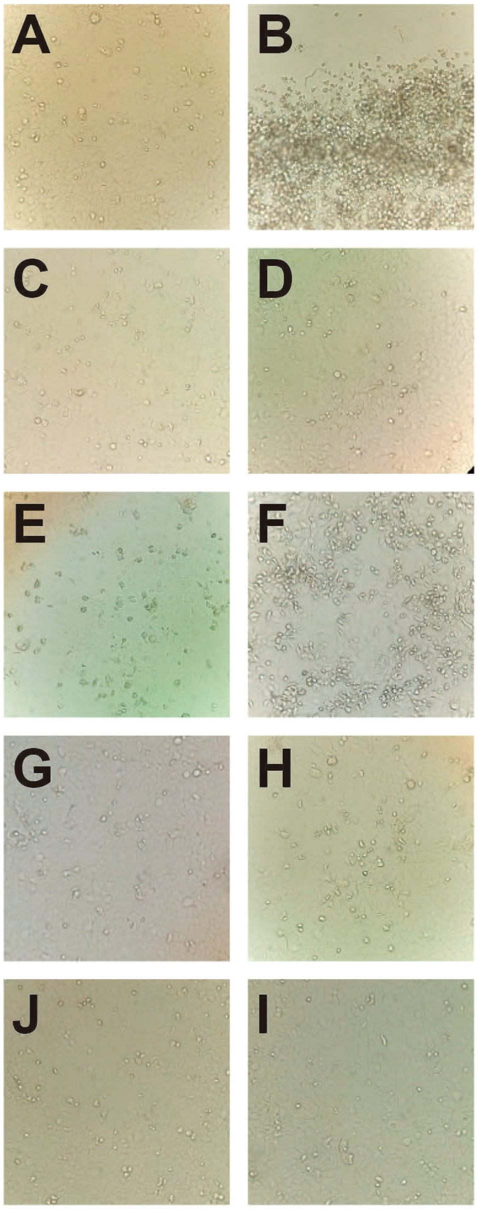

Figure S5. Morphological Effects of Individual Transition Metal Treatments on HaCat Cells. Optical Microscope Images at 200X of HaCat cells treated for 24h with (A) Negative Control, (B) Positive Control (DMSO 10%), (C) Ag 3.75µM, (D) Ag 15µM, (E) Co 400µM, (F) Co 750µM, (G) Cu 80µM, (H) Cu 300µM, (I) Zn 31.25µM, (J) Zn 125µM.

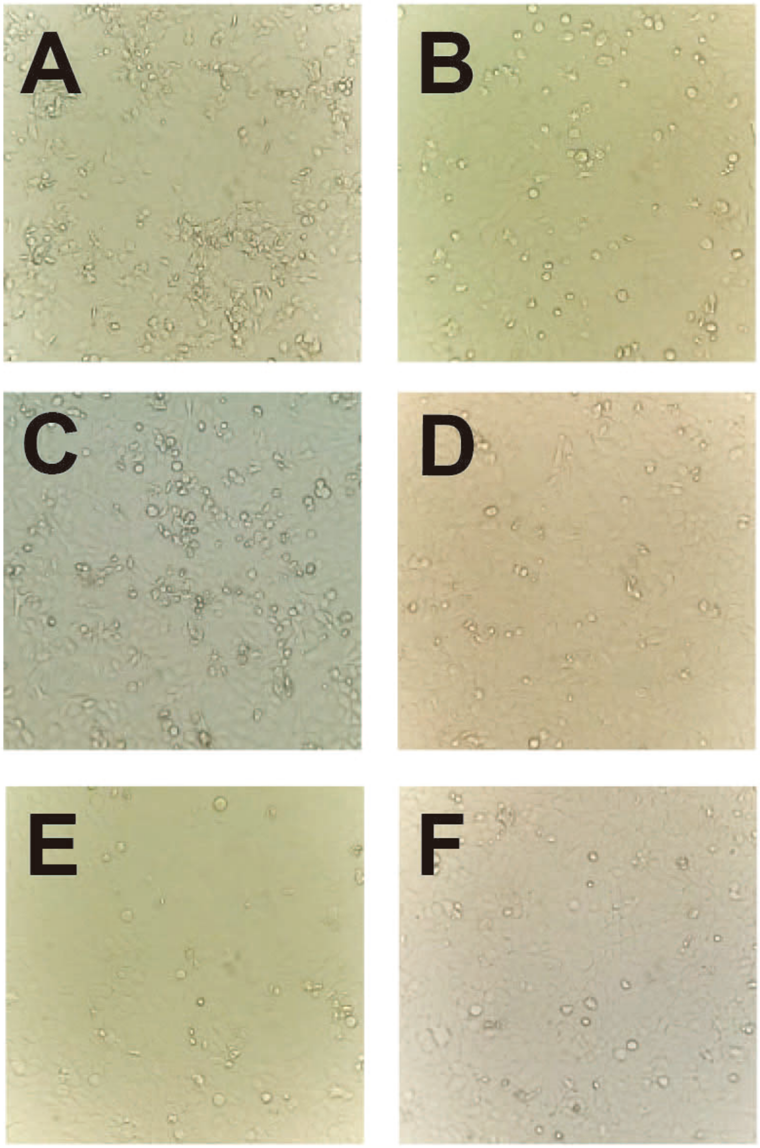

Figure S6. Morphological Effects of STMCs on HaCat Cells. Optical Microscope Images at 200X of HaCat cells treated for 24h with (A) Ag 7.5µM and Cu 300µM, (B) Ag 7.5µM and Cu 80µM, (C) Ag 15µM and Co 750µM, (D) Ag 15µM and Co 400µM, (E) Ag^{+} 15µM and Zn 125µM, (F) Ag 15µM and Zn 62.5µM
